# Supplementary material for: Genetic switches designed for eukaryotic cells and controlled by serine integrases
Source: Commun Biol. 2020 May 22;3:255. doi: 10.1038/s42003-020-0971-8 (PMC7244727; doi:10.1038/s42003-020-0971-8)
Supplement: Supplementary file 1 — Supplementary Information [file 42003_2020_971_MOESM1_ESM.pdf]

Supplementary Information

Genetic switches designed for eukaryotic cells and controlled by serine integrases

Mayna S. Gomide[1,2,3,], Thais T. Sales[1,2,], Luciana R.C. Barros[4], Cintia G. Limia[4] Marco A. de Oliveira[1,2], Lilian H. Florentino[1], Leila M.G. Barros[1], Maria L. Robledo[4], Gustavo P.C. José[1], Mariana S.M. Almeida[1], Rayane N. Lima[1], Stevens K. Rehen[5,6], Cristiano Lacorte[1], Eduardo O. Melo[1,7], André M. Murad[1], Martín H. Bonamino[4,8*], Cintia M. Coelho[9*] and Elibio Rech[1*].

1 Brazilian Agriculture Research Corporation – Embrapa – Genetic Resources and Biotechnology – CENARGEN, Brasília, 70770917, DF, Brazil;

2 Department of Cell Biology, Institute of Biological Science, University of Brasília, Brasília, 70910900, DF, Brazil;

3 School of Medicine, Federal University of Juiz de Fora, Juiz de Fora, 36036900, MG, Brazil;

4 Molecular Carcinogenesis Program, Research Coordination, National Cancer Institute (INCA), Rio de Janeiro, 20231050, RJ, Brazil;

5 D'Or Institute for Research and Education (IDOR), Rio de Janeiro, 22.281-100, RJ, Brazil;

6 Institute of Biomedical Sciences, Federal University of Rio de Janeiro, Rio de Janeiro, 21941-902, RJ, Brazil;

7 Graduation Program in Biotechnology, Federal University of Tocantins, Gurupi, 77402-970, TO, Brazil;

8 Vice-Presidency of Research and Biological Collections (VPPCB), FIOCRUZ – Oswaldo Cruz Foundation Institute, Rio de Janeiro, 21040900, RJ, Brazil;

9 Department of Genetic and Morphology, Institute of Biological Science, University of Brasília, Brasília, 70910900, DF, Brazil;

* Corresponding authors (emails: elibio.rech@embrapa.br; cintiacoelhom@unb.br; and mbonamino@inca.gov.br).

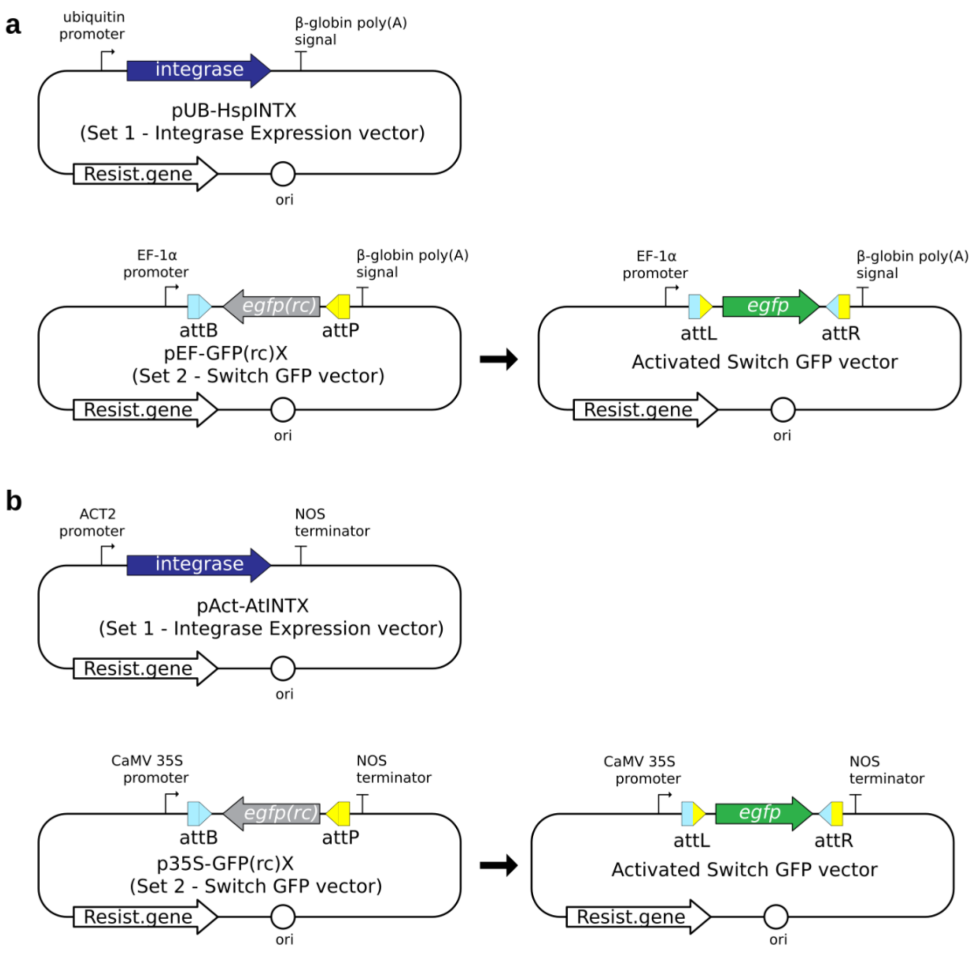

Supplementary Figure 1. Schematic representation of the vector sets synthesized for the eukaryotic genetic switches and the resulting activated vector. For human, bovine, and plant cells, two sets of plasmids were built to evaluate Int functionality. **a** For mammalian systems, one plasmid contains the human codon optimized sequence of Int X (X= 2, 4, 5, 7, 9, 13, phiC31 or Bxb1) under the control of the ubiquitin promoter and β-globin poly(A) signal terminator; this set was named the integrase expression vector (pIE) set, composed of plasmids named pUB-HspINTX. The other plasmid is composed of the reporter e*gfp* gene in the reverse complement (rc) orientation flanked by the recognition sites *attB*/*attP* of that particular Int, under the control of a different strong constitutive promoter, namely, the EF1alpha promoter, and the same terminator; this set was named the switch GFP vector (pSG), with plasmids named pEF-GFP(rc)X. **b** For plant protoplasts, the pIE vectors set contains the *A. thaliana* codon optimized sequence of Int X under the actin2 promoter and the NOS terminator, plasmids named pAct-AtINTX. The pSG vectors set has the e*gfp* sequence in the reverse complement orientation flanked by the recognition sites *attB*/*attP* of that particular Int, under the control of the CaMV 35S promoter and the same terminator, plasmids named p35S-GFP(rc)X (additional information on the plasmids is provided in Supplementary Table 1). Both plasmids of each set were used to cotransfect/cotransform the mammalian and plant systems. It was hypothesized that if a particular Int was functional, it would switch the e*gfp* coding sequence to the forward orientation, leading to EGFP expression and formation of the *attL*/*attR* sites (activated switch GFP vector).

INTEGRASE	NLS
INT_02	^{158}RTTRLNAKRGGAHGPVPDGYKRRYPD^{184} [6.0]
INT_04	^{260}RERQRRRLGIEENHYTIPFQAKYMLSKFLRC^{290} [5.9]
INT_05	
INT_07	^{131}RENLAERVKFGIEQMIDEGKKPGGHSPYGYKFDKD^{165} [5.4]
INT_09	^{13}EQKEKGHSIEEQERKLRAYSDINDWKIHKVY^{43} [5.3]
INT_13	^{283}VNRFIKKRKDGTEYC^{297} [8.5]
Bxb1	^{52}PFDRKRRPNL^{61} [5.0]
PhiC31	^{221}REIKTHKHLPFKPGSQAAIHPGSITGLCKRMDAD^{254} [4.8]

Supplementary Figure 2. *In silico* prediction of the nuclear localization signal (NLS) for the Int coding sequences. The NLS was predicted using NLS Mapper (available at http://nls-mapper.iab.keio.ac.jp/cgi-bin/NLS_Mapper_form.cgi, accessed in 10/08/2018) with an intermediate cut-off score (5.0). The positions of the first and last amino acids of the predicted NLS sequence are denoted as superscripted numbers. The basic amino acids arginine (R) and lysine (K) are highlighted in cyan. Scores are indicated in brackets.

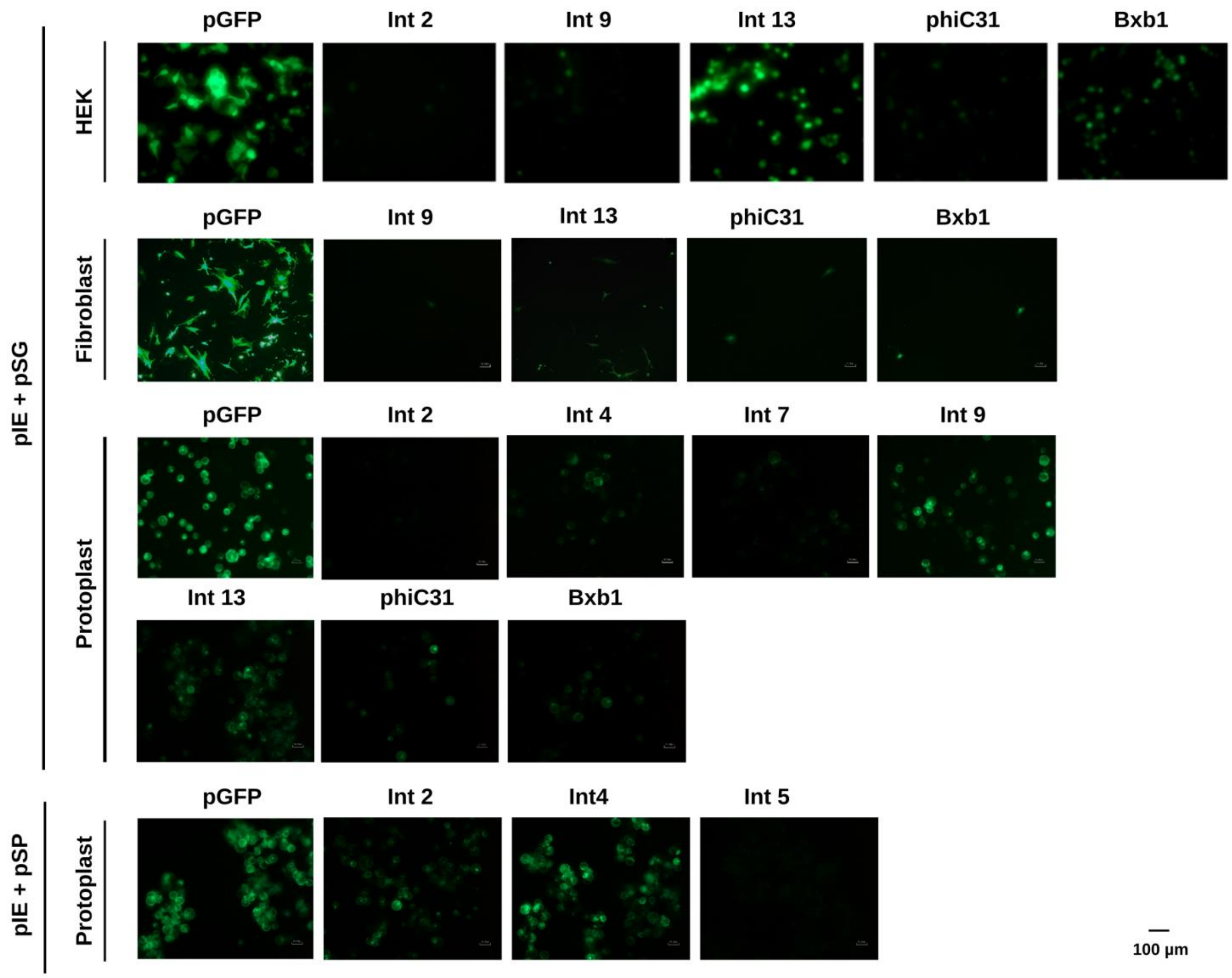

Supplementary Figure 3. Representative EGFP fluorescence images of the three model eukaryotic cell systems cotransfected/cotransformed with integrase expression (pIE) and switch GFP or promoter (pSG or pSP) vectors. HEK 293T cells, bovine fibroblasts and plant protoplasts were observed using an Axiovert 135M (Carl Zeiss) fluorescence microscope. The images were acquired using an attached DS-Ri1 digital camera (Nikon) and the capture software Nikon Digital Sight DS-L3 (Nikon) under a UV light with filter set 15 (Carl Zeiss). Excitation: BP 546; beam splitter: FT 580; emission: LP 590. For each cell model, all images were acquired with the same acquisition setting.

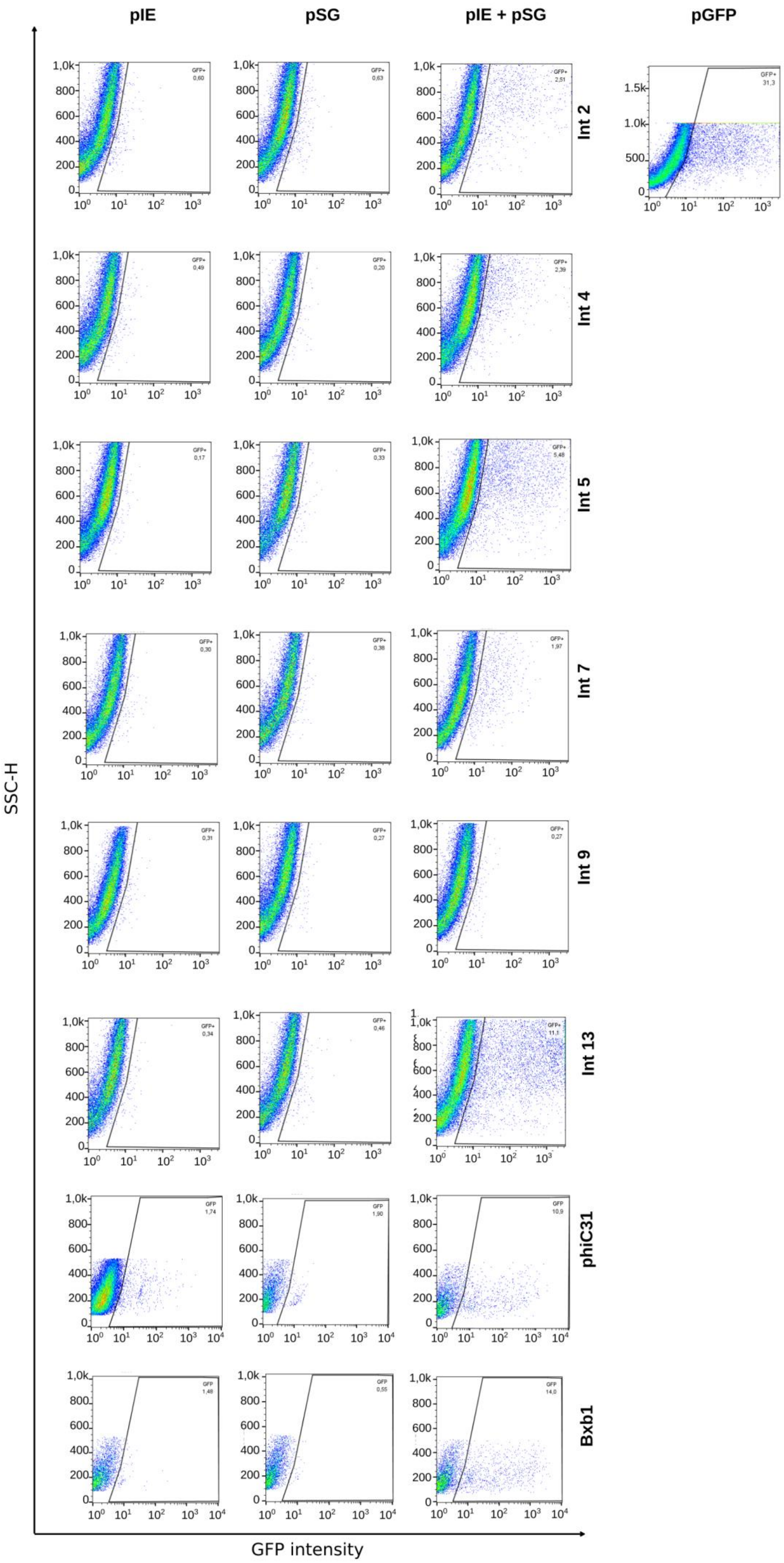
pIE
pSG
pIE + pSG
pGFP
Int 2
Int 4
Int 5
Int 7
Int 9
Int 13
phiC31
Bxb1
SSC-H
GFP intensity

Supplementary Figure 4. EGFP fluorescence determined by flow cytometry analysis of HEK 293T cells. Representative scatter plots are shown indicating the EGFP fluorescence-emitting population in the gate. Experimental groups were analyzed after 48 h. The integrase expression vectors (pIE) of the Ints 2, 4, 5, 7, 9, 13, phiC31, and Bxb1 or the switch GFP vectors (pSG) containing the *egfp* gene in reverse complement orientation flanked by *attB/attP* sites of the Ints 2, 4, 5, 7, 9, 13, phiC31, and Bxb1 indicate negative controls. pIE + pSG indicates the cells cotransfected with integrase expression and switch GFP vectors (test condition). Positive control cells were transfected with the pT3-Neo-EF1α-GFP plasmid containing the *egfp* sequence in the forward orientation (pGFP).

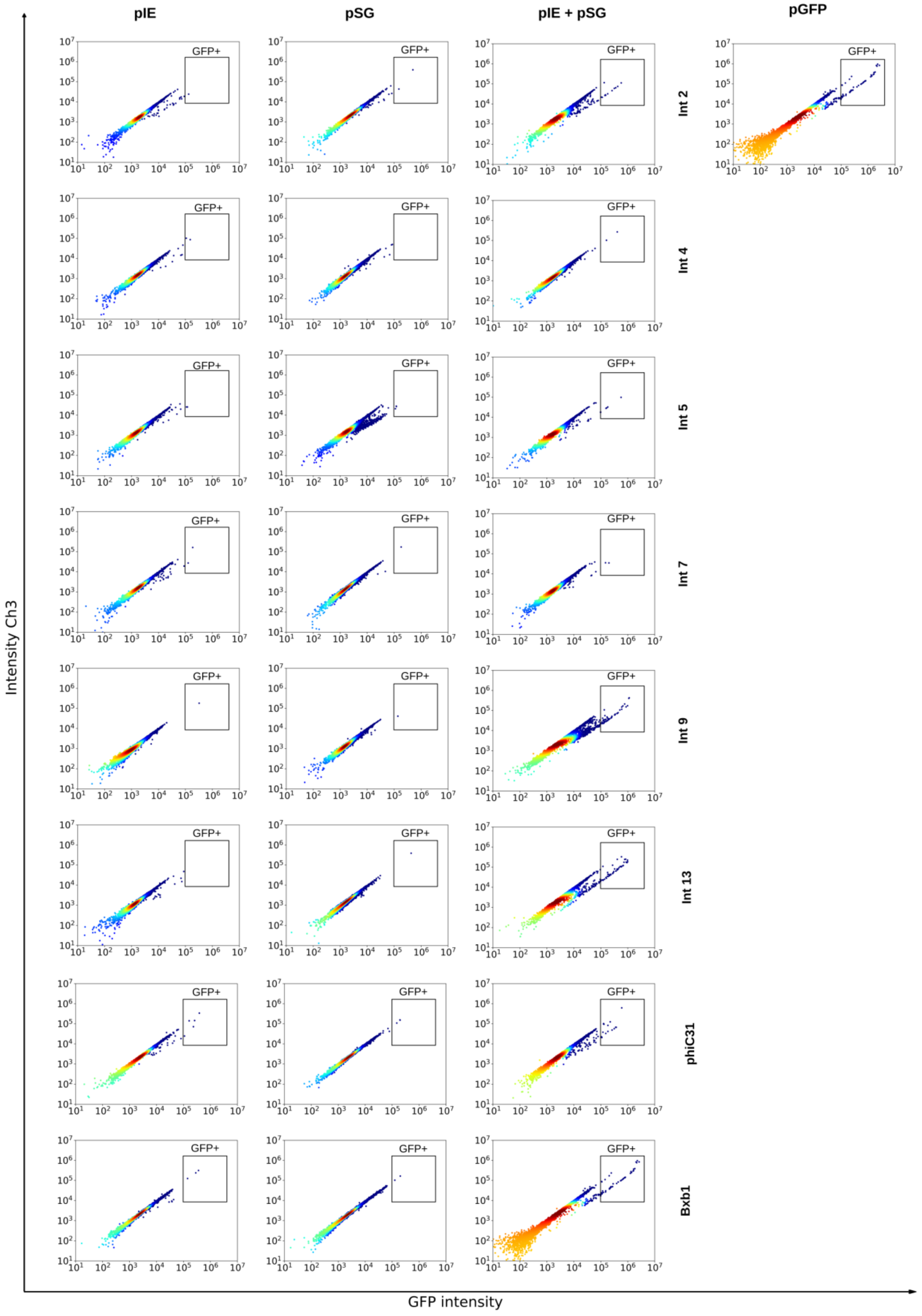

Supplementary Figure 5. EGFP fluorescence determined by flow cytometry analysis of bovine fibroblasts. Representative scatter plots are shown indicating the EGFP fluorescence-emitting population in the gate. Experimental groups were analyzed after 48 h. Negative control cells were cotransfected with one of the plasmids from the integrase expression vectors (pIE) of the Ints 2, 4, 5, 7, 9, 13, phiC31, and Bxb1 or one of the switch GFP vectors (pSG) containing the *egfp* gene in reverse complement

orientation flanked by *attB/attP* sites of the Ints 2, 4, 5, 7, 9, 13, phiC31, and Bxb1 plus a mock plasmid. pIE + pSG indicates the cells cotransfected with integrase expression and switch GFP vectors (test condition). Positive control cells were cotransfected with the pEF-GFP plasmid containing the *egfp* sequence in the forward orientation (pGFP) plus a mock plasmid.

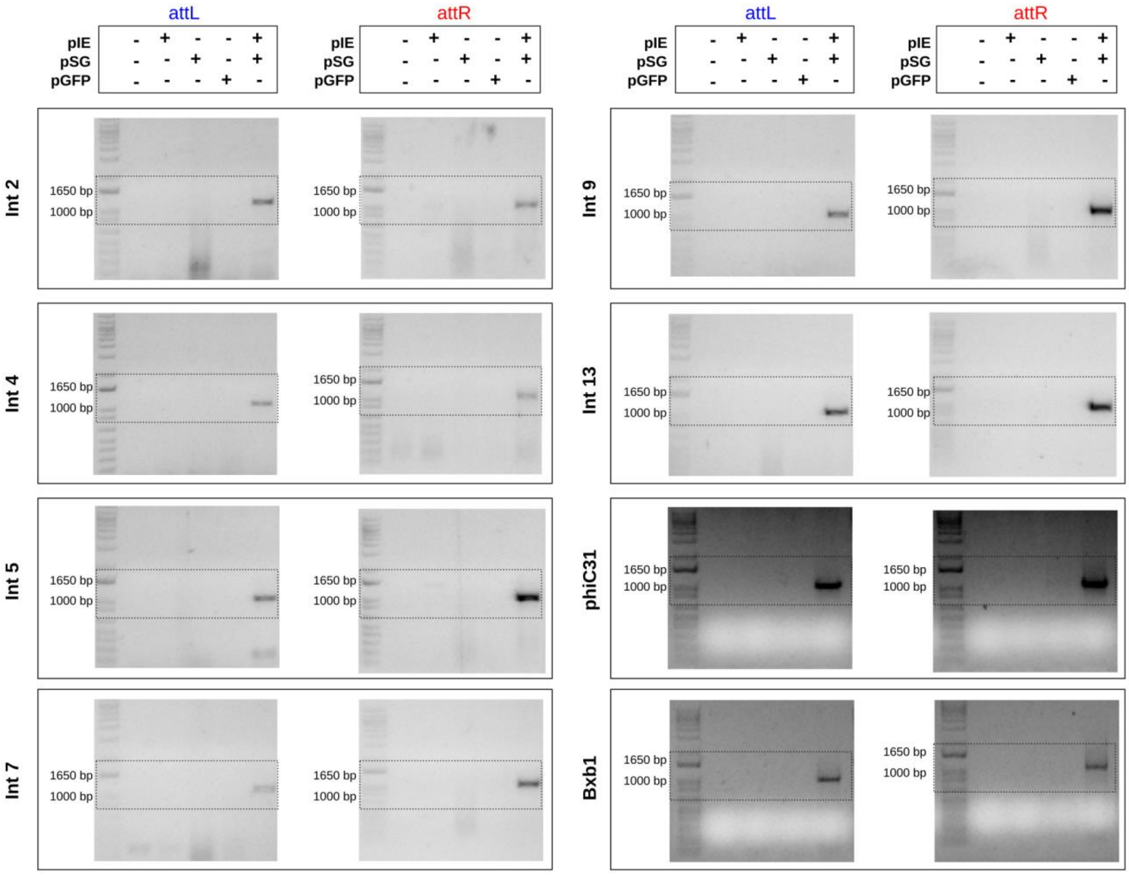

Supplementary Figure 6. HEK 293T cells uncropped PCR gel images. Amplicons obtained using two specific primer sets to verify *attL* (blue) and *attR* (red) formation after *egfp* flipping as shown in the main text Fig. 2c.

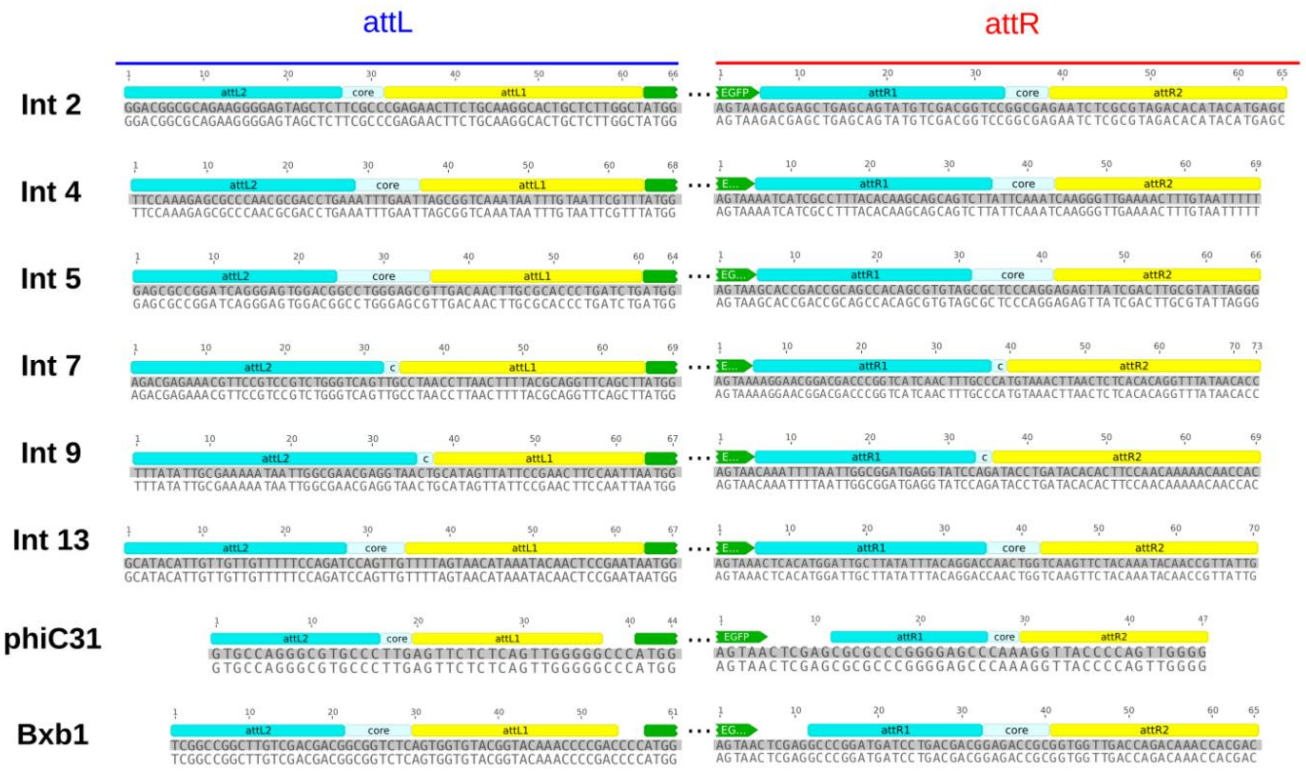

Supplementary Figure 7. Representative sequence reads showing the *attL* and *attR* sites obtained after Int activity in HEK 293T cells compared to the predicted sequences (grey highlighted). *attL*1 and *attR*1 correspond to the flipped *attP* and *attB* parts, respectively. *attL*2 and *attR*2 correspond to the previous *attB* and *attP* parts, respectively. Additional information in Supplementary Table 3 and Supplementary Data 1.

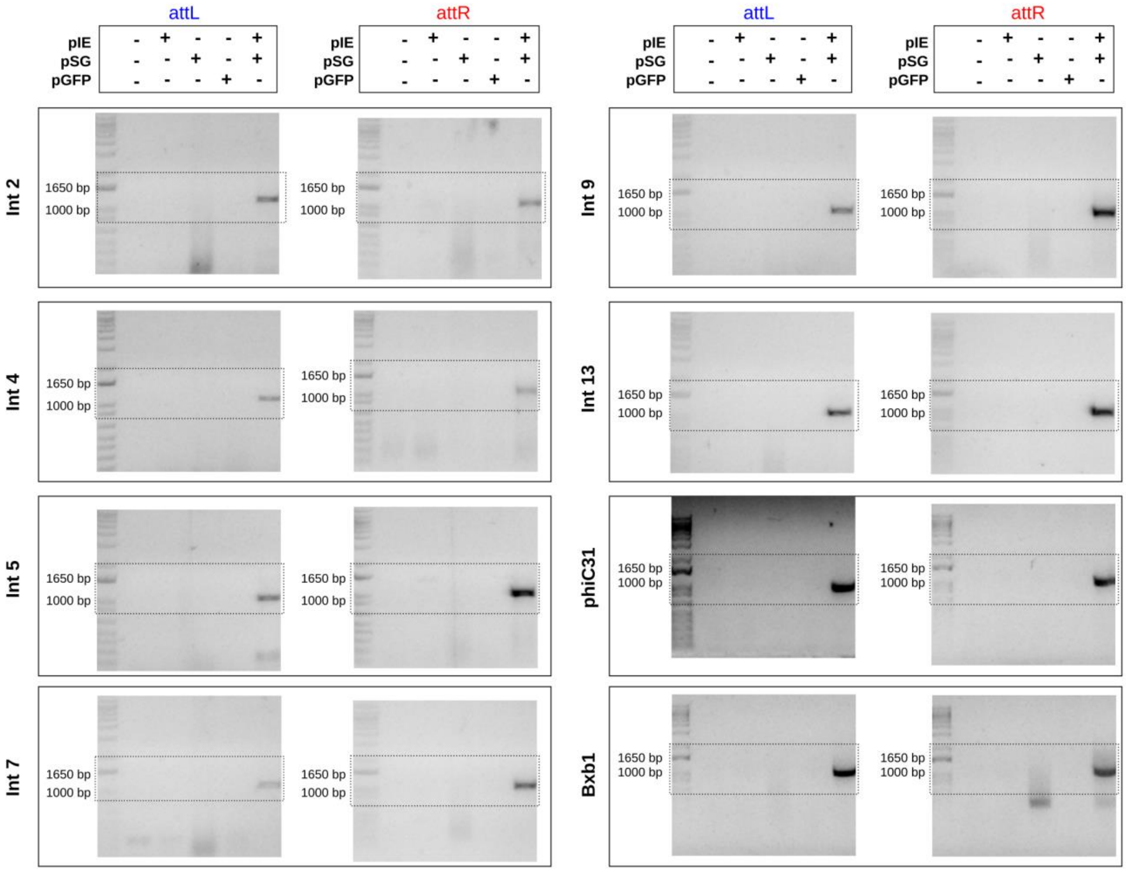

Supplementary Figure 8. Bovine fibroblasts uncropped PCR gel images. Amplicons obtained using two specific primer sets to verify *attL* (blue) and *attR* (red) formation after *egfp* flipping as shown in the main text Fig. 3c.

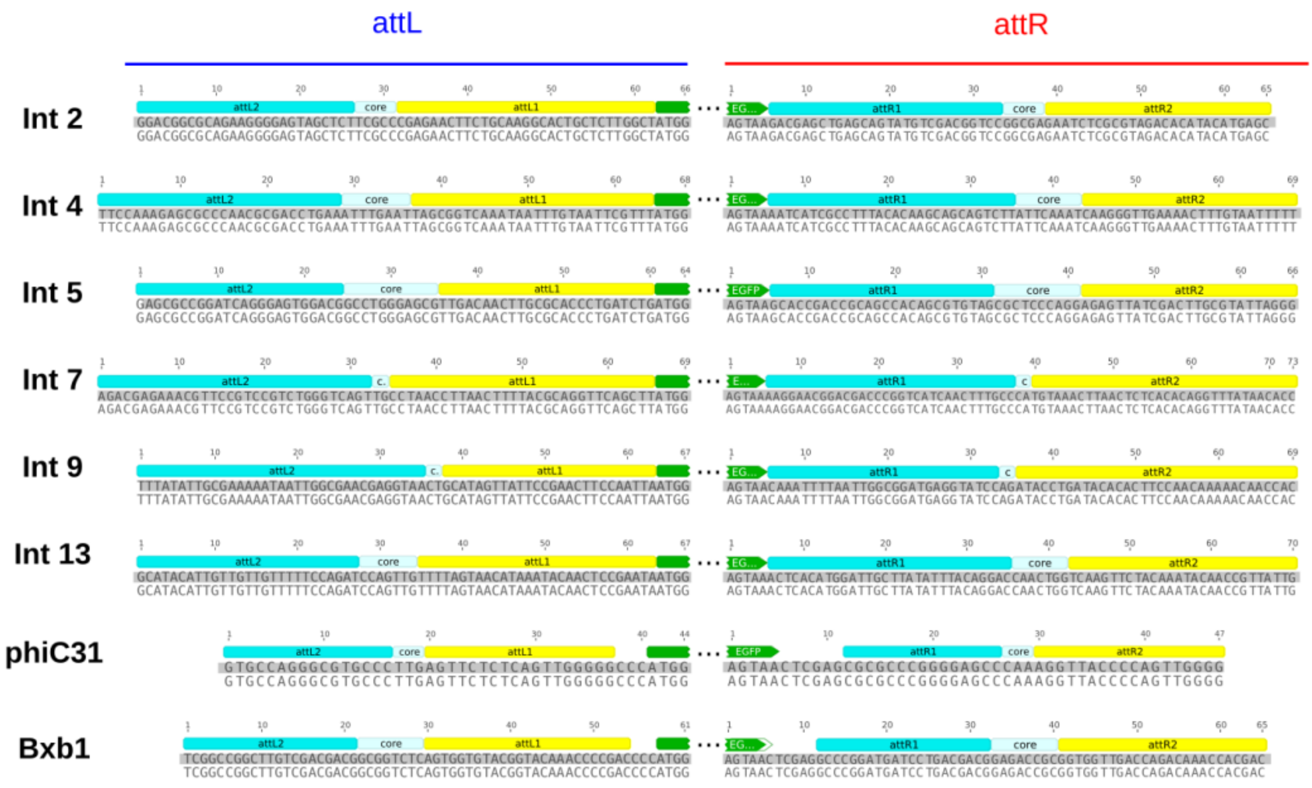

Supplementary Figure 9. Representative sequence reads showing the *attL* and *attR* sites obtained after Int activity in bovine fibroblasts compared to the predicted sequences (grey highlighted). *attL1* and *attR1* correspond to the flipped *attP* and *attB* parts, respectively. *attL2* and *attR2* correspond to the previous *attB* and *attP* parts, respectively. Additional information in Supplementary Table 3 and Supplementary Data 2.

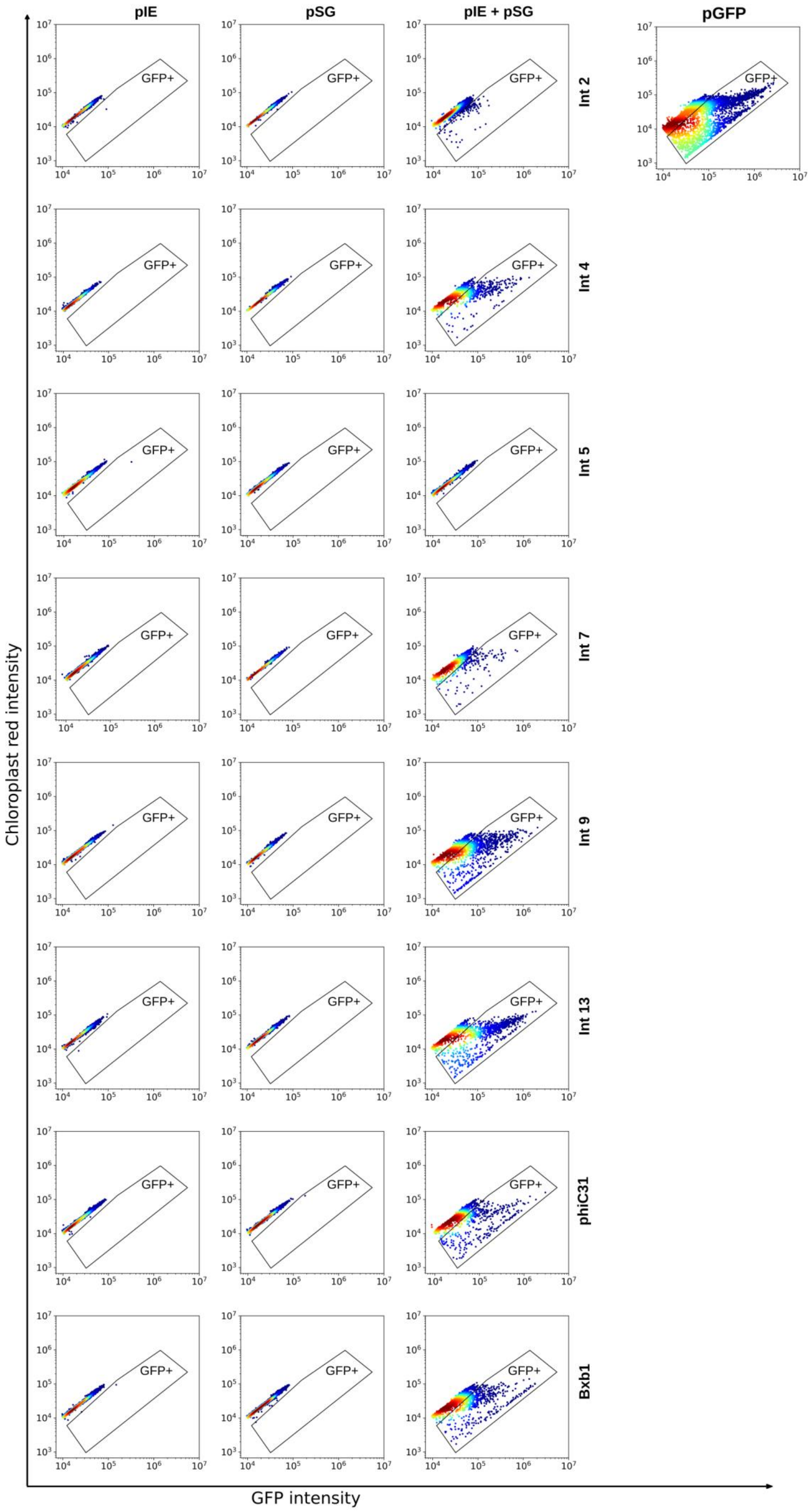

pIE
pSG
pIE + pSG
pGFP
Int 2
Int 4
Int 5
Int 7
Int 9
Int 13
phiC31
Bxb1
GFP+
Chloroplast red intensity
GFP intensity

Supplementary Figure 10. EGFP fluorescence determined by flow cytometry analysis of *A. thaliana* protoplasts. Representative scatter plots are shown indicating the EGFP fluorescence-emitting population in the gate. Experimental groups were analyzed after 24 h. Negative control cells were cotransformed with one of the plasmids from the integrase expression vectors (pIE) of the Ints 2, 4, 5, 7, 9, 13, phiC31, and Bxb1 or the switch GFP vectors (pSG) containing the *egfp* gene in reverse complement orientation flanked by *attB/attP* sites of the Ints 2, 4, 5, 7, 9, 13, phiC31, and Bxb1 plus a mock plasmid. pIE+ pSG indicates the cells cotransformed with integrase expression and switch GFP vectors (test condition). Positive control cells were cotransformed with the pCaMV35S-GFP plasmid containing the *egfp* sequence in the forward orientation (pGFP) plus a mock plasmid.

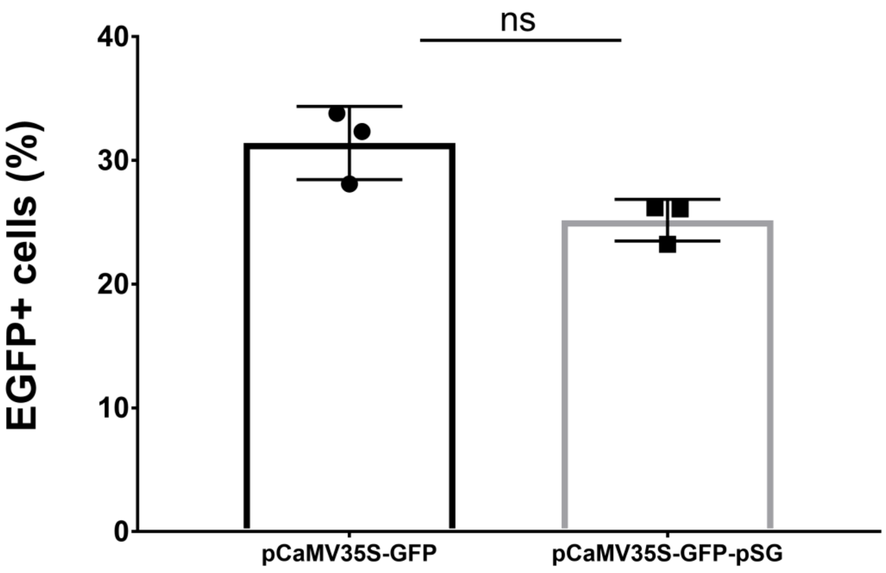

Supplementary Figure 11. EGFP-expressing cell percentages of the positive control pCaMV35S-GFP vector and pCaMV35S-GFP-pSG vector. pCaMV35S-GFP (pGFP) has a CaMV 35S promoter with some SNPs compared with the CaMV 35S promoter used in the switch GFP vectors (pSG). Then, the CaMV 35S promoter from pSG was cloned, replacing the pCaMV35S-GFP promoter, resulting in the pCaMV35S-GFP-pSG plasmid. Protoplasts were transformed with both plasmids separately, and flow cytometry analysis showed that the percentage of EGFP-expressing cell populations obtained with the two constructs did not result in statistically significant differences. The statistical analysis was performed in GraphPad Prism 7, applying a paired T test. p value=0.1277. Assays were performed in five or six technical replicates and in three biologically independent experiments.

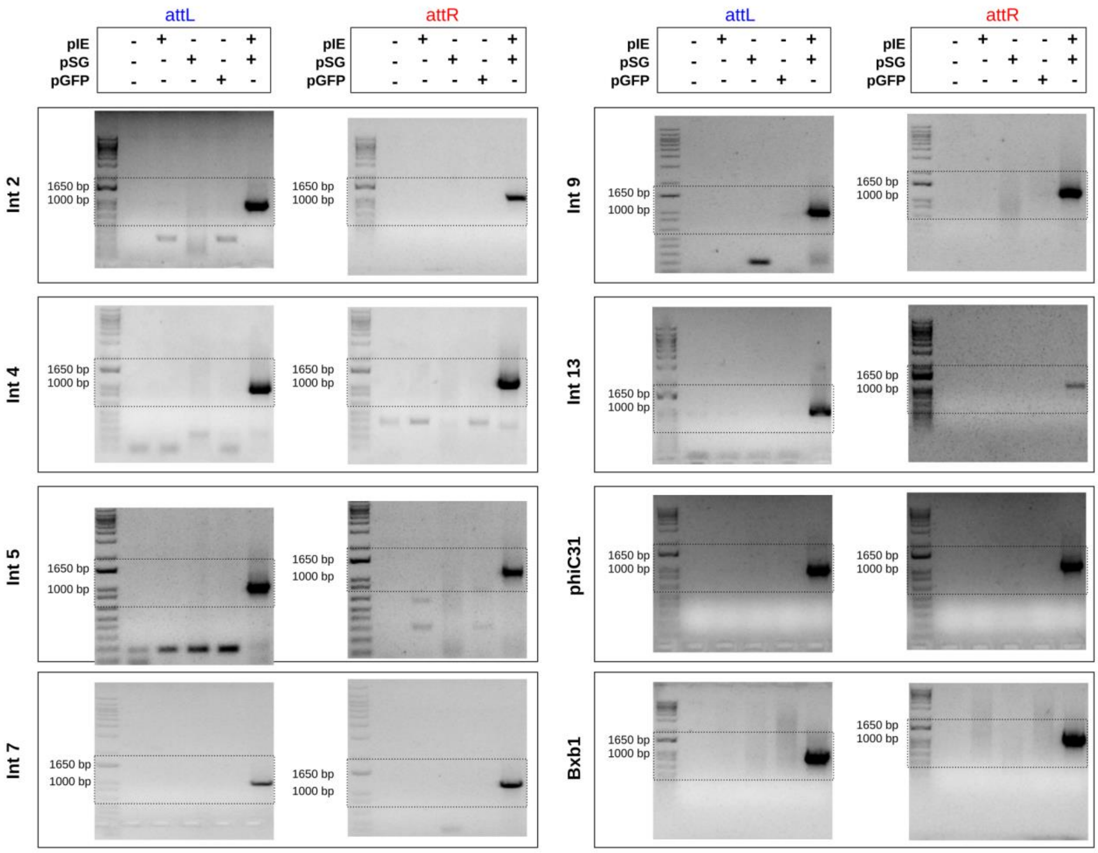

Supplementary Figure 12. *A. thaliana* protoplasts uncropped PCR gel images. Amplicons obtained using two specific primer sets to verify *attL* (blue) and *attR* (red) formation after *egfp* flipping as shown in the main text Fig. 4c.

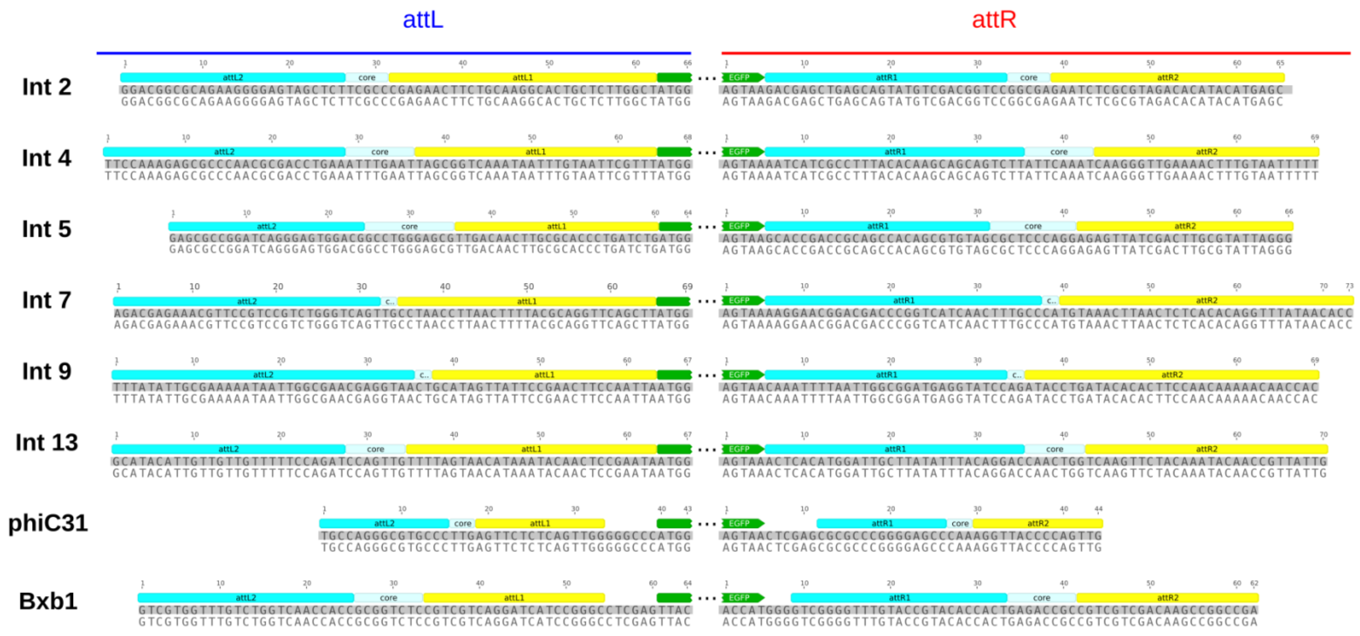

Supplementary Figure 13. Representative sequence reads showing the *attL* and *attR* sites obtained after Int activity in *A. thaliana* protoplasts compared to the predicted sequences (grey highlighted). *attL*1 and *attR*1 correspond to the flipped *attP* and *attB* parts, respectively. *attL*2 and *attR*2 correspond to the previous *attB* and *attP* parts, respectively. Additional information in Supplementary Table 3 and Supplementary Data 3.

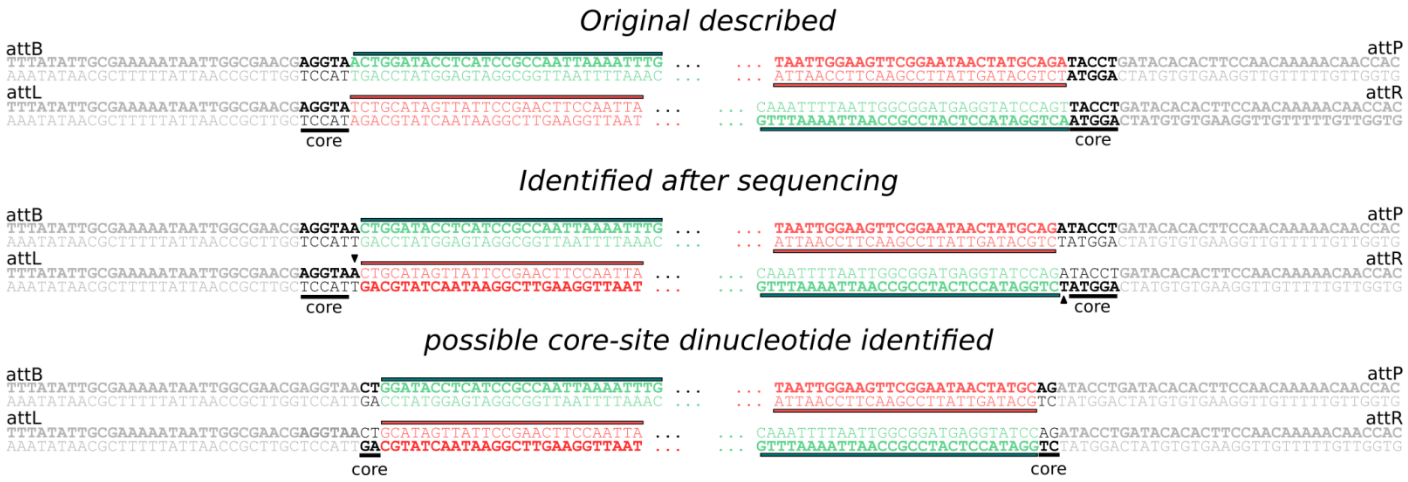

Supplementary Figure 14. Int 9 core-site differences compared to the original sequences described[1]. The Int 9 sequence alignments indicated one additional nucleotide near to core-site (black arrow top). Considering the integrase functional mechanism that leads to a rotational and religation of half part of the recognition *attB/P* sites forming the *attL/attR* sites (green and red parts), a possible solution was to consider CT nucleotide as Int9 core-site.

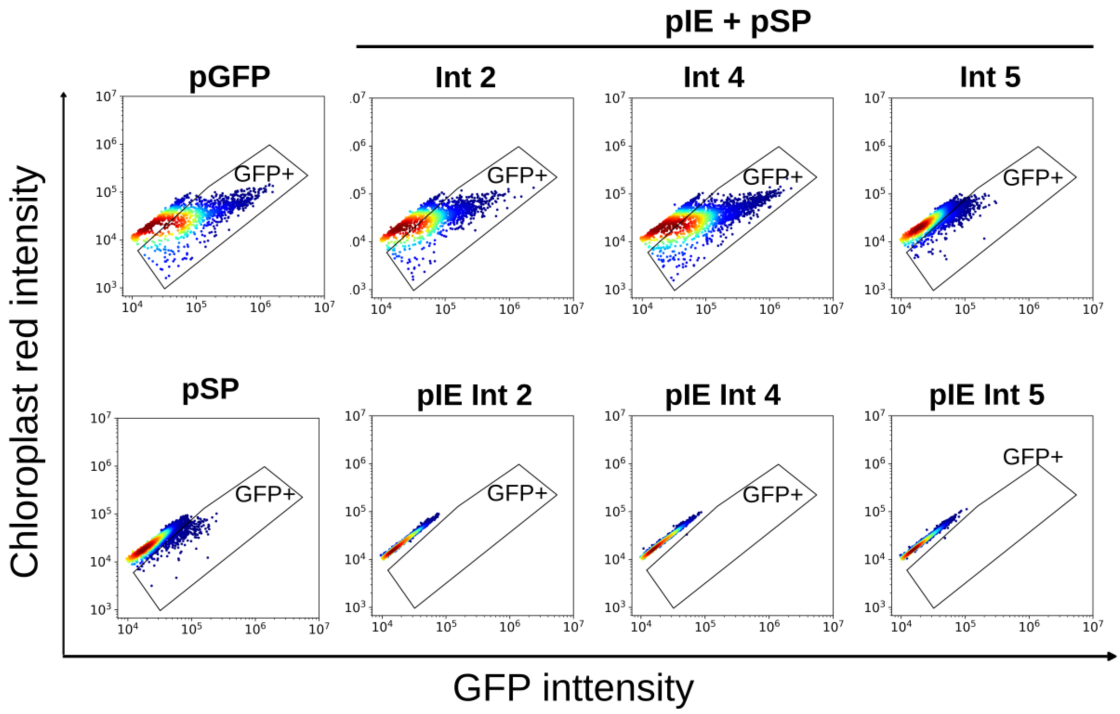

Supplementary Figure 15. EGFP fluorescence determined by flow cytometry analysis of *A. thaliana* protoplasts with the switch promoter system. Representative scatter plots are shown indicating the EGFP fluorescence-emitting population in the gate. Experimental groups were analyzed after 24 h. Negative control cells were cotransformed with one of the plasmids from the integrase expression vectors (pIE) of Ints 2, 4 and 5 or with the switch Promoter vector (pSP) containing the CaMV 35S promoter in reverse complement orientation flanked by *attB/attP* sites of Ints 2, 4 and 5 in tandem plus a mock plasmid. pIE+ pSP indicates the cells cotransformed with integrase expression vectors and the switch promoter vector (test condition). Positive control cells were cotransformed with the pCaMV35S-GFP plasmid containing the CaMV 35S promoter in the forward orientation (pGFP) plus a mock plasmid.

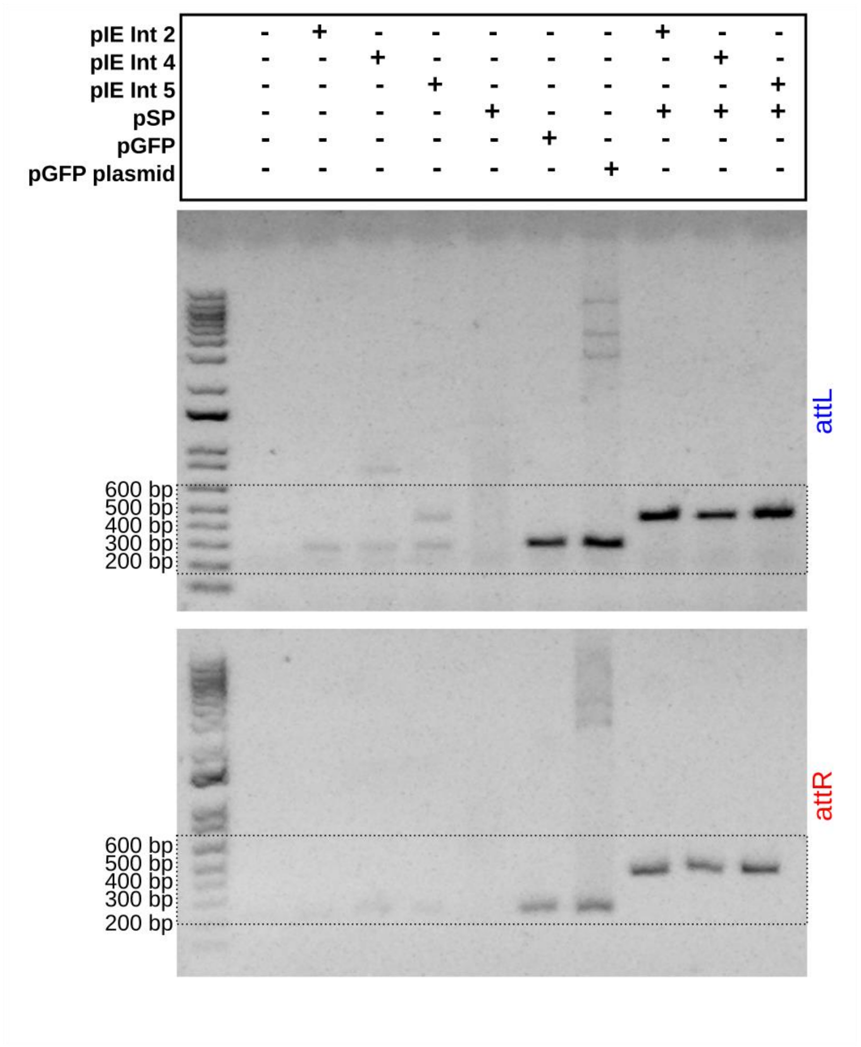

Supplementary Figure 16. *A. thaliana* protoplasts uncropped PCR gel images. Amplicons obtained using two specific primer sets to verify *attL* (blue) and *attR* (red) formation after CaMV 35S promoter flipping as shown in the main text Fig. 5d.

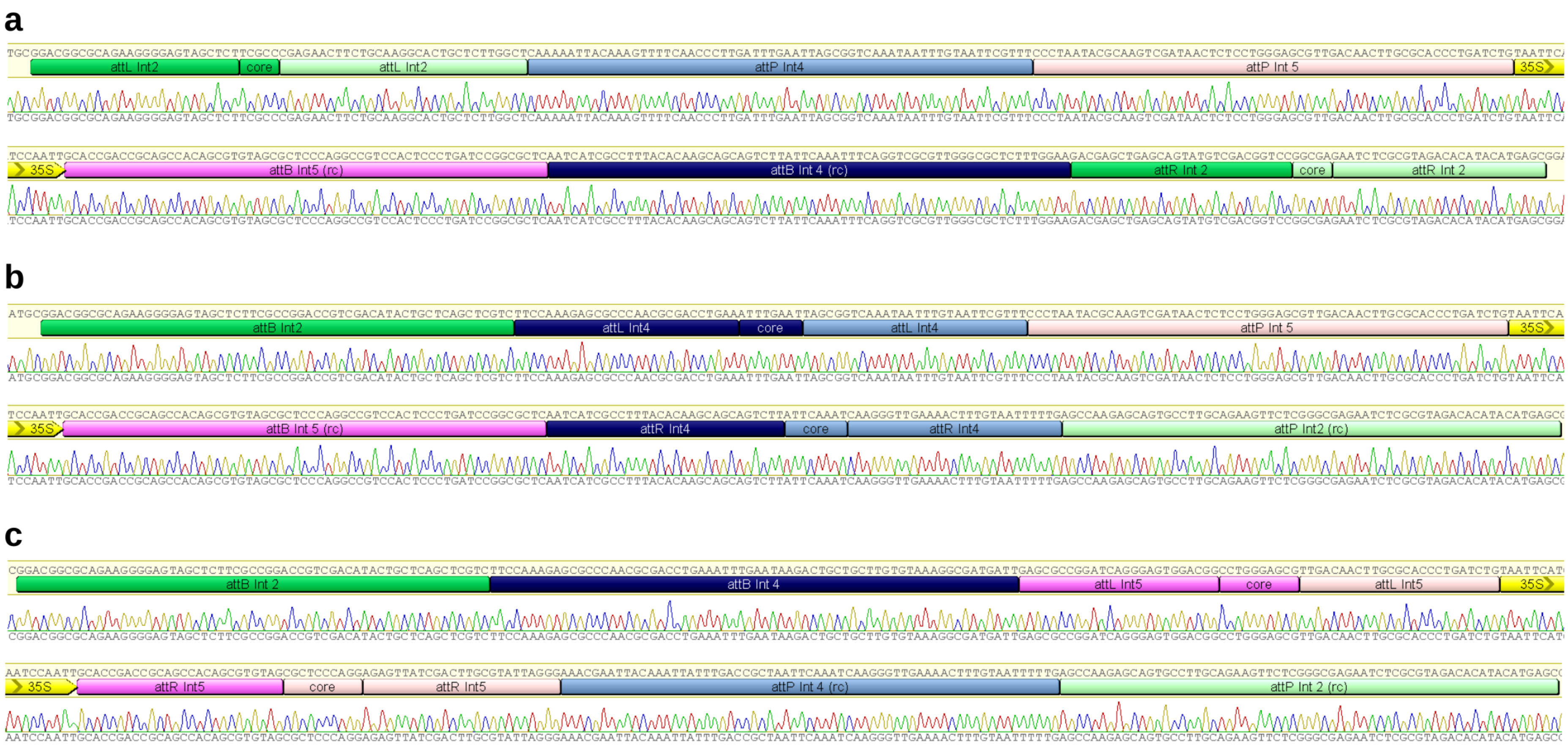

Supplementary Figure 17. Representative chromatograms showing the recognition site sequences obtained after Int activity in the switch promoter vector (pSP). **a** Recognition sites after Int 2 activity, resulting in *attL*/*attR* Int 2 sites and *attB/attP* Ints 4 and 5 sites in the expected positions. **b** Recognition sites after Int 4 activity, resulting in *attL*/*attR* Int 4 sites and *attB/attP* Ints 2 and 5 sites in the expected positions. **c** Recognition sites after Int 5 activity, resulting in *attL*/*attR* Int 5 sites and *attB/attP* Ints 2 and 4 sites in the expected positions. The core represents the region where cleavage occurred (crossover site). Additional information is provided in Supplementary Data 4.

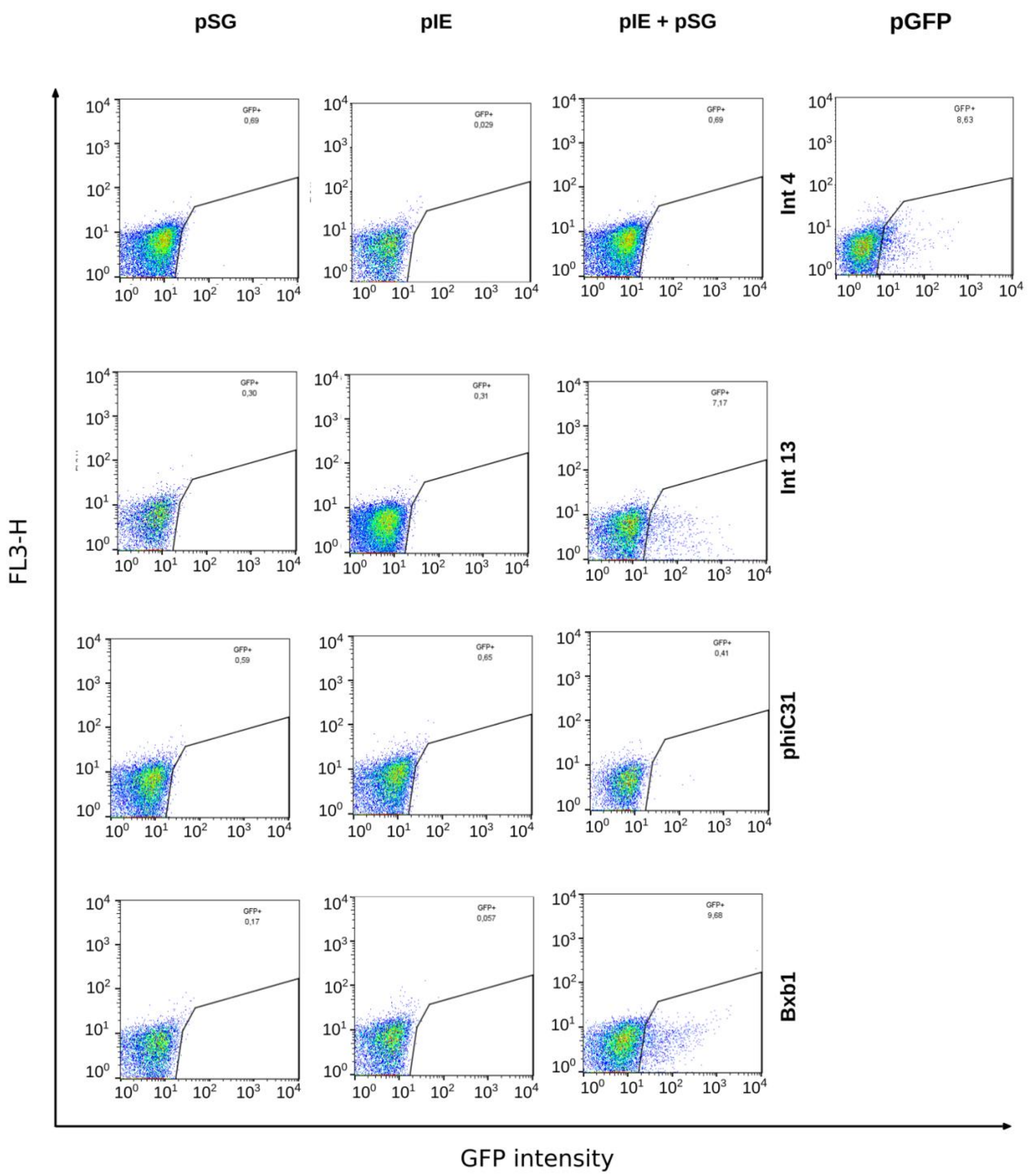

Supplementary Figure 18. EGFP fluorescence determined by flow cytometry analysis of PBMCs. Representative scatter plots are shown, indicating the EGFP fluorescence-emitting population in the gate. Experimental groups were analyzed after 48 h. Cells electroporated with the integrase expression vectors (pIE) of the Ints 4, 13, phiC3,1 and Bxb1 or the switch GFP vectors (pSG) containing the *egfp* sequence in reverse complement orientation flanked by *attB/attP* sites of the Ints 4, 13, phiC31, and Bxb1 indicate negative controls. pIE + pSG indicates the cells coelectroporated with integrase expression and switch GFP vectors (test condition). Positive control cells were transfected with the pT3-Neo-EF1α-GFP plasmid containing the *egfp* sequence in the forward orientation (pGFP).

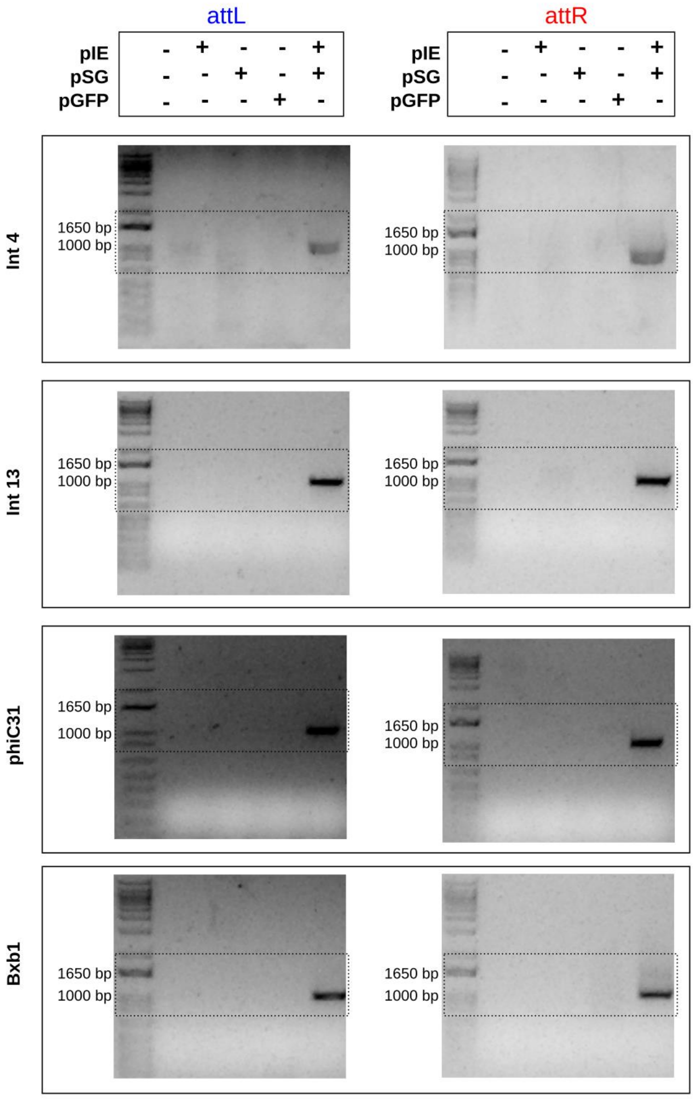

Supplementary Figure 19. PBMCs uncropped PCR gel images. Amplicons obtained using two specific primer sets to verify *attL* (blue) and *attR* (red) formation after *egfp* flipping as shown in the main text Fig. 6c.

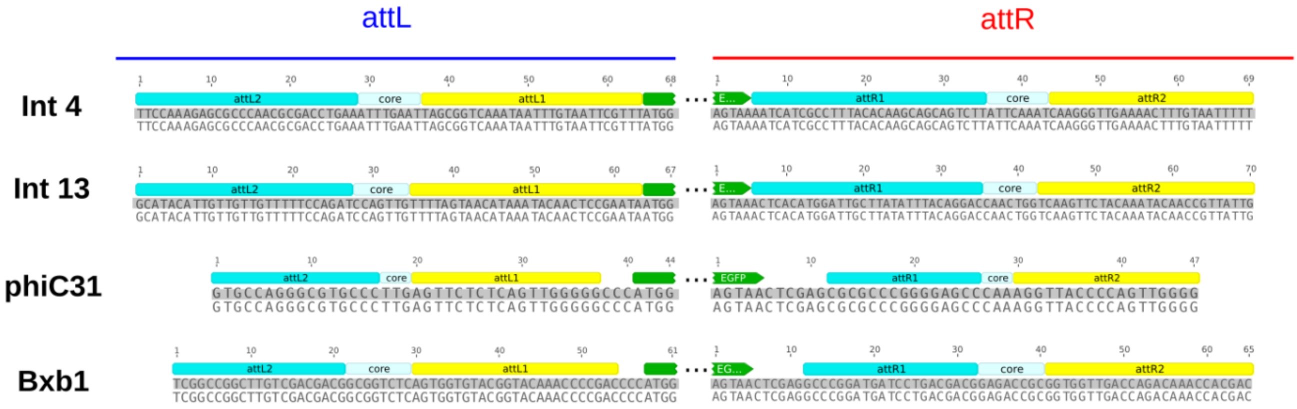

Supplementary Figure 20. Representative sequence reads showing the *attL* and *attR* sites obtained after Int activity in PBMCs compared to the predicted sequences (grey highlighted). *attL1* and *attR1* correspond to the flipped *attP* and *attB* parts, respectively. *attL2* and *attR2* correspond to the previous *attB* and *attP* parts, respectively. Additional information in Supplementary Table 6 and Supplementary Data 5.

Supplementary Table 1. Addgene accession numbers of all plasmids used in this study.

Vector set	Plasmid name	Addgene accession number
pSG	INCTbiosyn-pEF-GFP(rc)2	#127504
	INCTbiosyn-pEF-GFP(rc)4	#127505
	INCTbiosyn-pEF-GFP(rc)5	#127506
	INCTbiosyn-pEF-GFP(rc)7	#127507
	INCTbiosyn-pEF-GFP(rc)9	#127508
	INCTbiosyn-pEF-GFP(rc)13	#127509
	INCTbiosyn-pEF-GFP(rc)phiC31	#127510
	INCTbiosyn-pEF-GFP(rc)Bxb1	#127511
pIE	INCTbiosyn-pUB-HspINT2	#127512
	INCTbiosyn-pUB-HspINT4	#127513
	INCTbiosyn-pUB-HspINT5	#127514
	INCTbiosyn-pUB-HspINT7	#127515
	INCTbiosyn-pUB-HspINT9	#127516
	INCTbiosyn-pUB-HspINT13	#127517
	INCTbiosyn-pUB-HspINTphiC31	#127518
	INCTbiosyn-pUB-HspINTBxb1	#127519
pSP	INCTbiosyn-p35S(rc)2_4_5-GFP	#127520
pSG	INCTbiosyn-p35S-GFP(rc)2	#127521
	INCTbiosyn-p35S-GFP(rc)4	#127522
	INCTbiosyn-p35S-GFP(rc)5	#127523
	INCTbiosyn-p35S-GFP(rc)7	#127524
	INCTbiosyn-p35S-GFP(rc)9	#127525
	INCTbiosyn-p35S-GFP(rc)13	#127526
	INCTbiosyn-p35S-GFP(rc)phiC31	#127527
	INCTbiosyn-p35S-GFP(rc)Bxb1	#127528
pIE	INCTbiosyn-pAct-AtINT2	#127529
	INCTbiosyn-pAct-AtINT4	#127530
	INCTbiosyn-pAct-AtINT5	#127531
	INCTbiosyn-pAct-AtINT7	#127532
	INCTbiosyn-pAct-AtINT9	#127533
	INCTbiosyn-pAct-AtINT13	#127534
	INCTbiosyn-pAct-AtINTphiC31	#127535
	INCTbiosyn-pAct-AtINTBxb1	#127536

Supplementary Table 2. Summary of the statistical analysis of the EGFP-positive cell percentages obtained by flow cytometry assays with the switch GFP system in HEK 293T cells, bovine fibroblasts and plant protoplasts.

	Cell type	Int	Mean ± sd				Assays Repetition (n)	Kruskal-Wallis statistics (χ^2)	df	p-value
			pGFP	pIE + pSG	pIE	pSG				
Switch GFP system	HEK 293T	Int 2	21.32 ± 11.84[a]	1.29 ± 1.09[b]	2.93 ± 3.44[b]	0.33 ± 0.22[c]	3	20.30	3	0.00015
		Int 4	21.32 ± 11.84[a]	1.33 ± 1.27[b]	0.36 ± 0.10[b]	0.17 ± 0.08[c]	3	24.94	3	1.59×10^{-5}
		Int 5	21.32 ± 11.84[a]	2.52 ± 3.29[b]	0.35 ± 0.19[b]	0.30 ± 0.14[b]	3	17.51	3	0.00055
		Int 7	21.32 ± 11.84[a]	0.93 ± 0.83[b]	0.36 ± 0.18[b]	0.47 ± 0.27[b]	3	18.31	3	0.00038
		Int 9	21.32 ± 11.84[a]	0.48 ± 0.24[b]	0.21 ± 0.08[c]	0.58 ± 0.41[b]	3	21.82	3	7.13×10^{-5}
		Int 13	21.32 ± 11.84[a]	7.07 ± 5.31[b]	0.44 ± 0.30[c]	0.39 ± 0.32[c]	3	24.79	3	1.71×10^{-5}
		phiC31	21.32 ± 11.84[a]	10.79 ± 3.72[a]	2.36 ± 2.00[b]	1.37 ± 1.22[b]	3	20.96	3	0.00011
		Bxb1	21.32 ± 11.84[a]	16.02 ± 7.25[a]	1.82 ± 1.98[b]	2.30 ± 2.74[b]	3	20.29	3	0.00015
	Bovine Fibroblast	Int 2	1.80 ± 0.49[a]	0.12 ± 0.04[b]	0.04 ± 0.03[c]	0.02 ± 0.03[c]	3	29.37	3	1.87×10^{-6}
		Int 4	1.80 ± 0.49[a]	0.03 ± 0.03[b]	0.02 ± 0.02[bc]	0.01 ± 0.01[c]	3	23.04	3	3.97×10^{-5}
		Int 5	1.44 ± 0.79[a]	0.04 ± 0.02[b]	0.01 ± 0.01[c]	0.02 ± 0.03[c]	3	23.57	3	3.08×10^{-5}
		Int 7	1.44 ± 0.79[a]	0.03 ± 0.02[b]	0.02 ± 0.02[b]	0.02 ± 0.01[b]	3	20.95	3	0.00011
		Int 9	4.51 ± 3.99[a]	0.28 ± 0.12[b]	0.02 ± 0.02[c]	0.01 ± 0.01[c]	3	30.46	3	1.10×10^{-6}
		Int 13	4.51 ± 3.99[a]	0.74 ± 0.57[b]	0.02 ± 0.02[c]	0.01 ± 0.01[c]	3	29.19	3	2.04×10^{-6}
		phiC31	1.34 ± 0.48[a]	0.13 ± 0.03[b]	0.04 ± 0.04[c]	0.02 ± 0.01[d]	3	29.68	3	1.61×10^{-6}
		Bxb1	1.34 ± 0.48[a]	0.33 ± 0.12[b]	0.03 ± 0.02[c]	0.01 ± 0.01[d]	3	30.84	3	9.18×10^{-7}
	Protoplast	Int 2	30.95 ± 8.79[a]	2.71 ± 2.29[b]	0.00 ± 0.01[d]	0.02 ± 0.03[c]	5	52.31	3	2.57×10^{-11}
		Int 4	30.57 ± 4.31[a]	17.77 ± 3.70[b]	0.00 ± 0.00[c]	0.00 ± 0.00[c]	3	33.76	3	2.22×10^{-7}
		Int 5	23.08 ± 5.52[a]	0.14 ±0.13[b]	0.00 ± 0.00[c]	0.01 ± 0.02[c]	3	28.96	3	2.28×10^{-6}
		Int 7	27.91 ±10.99[a]	10.96 ± 3.92[b]	0.01 ± 0.03[c]	0.00 ± 0.00[c]	4	43.01	3	2.45×10^{-9}
		Int 9	28.44 ± 3.03[a]	16.44 ± 7.59[b]	0.01 ± 0.02[c]	0.00 ± 0.01[c]	3	31.33	3	7.24×10^{-7}
		Int 13	21.40 ± 9.12[a]	24.24 ± 9.98[a]	0.01 ± 0.02[b]	0.01 ± 0.02[b]	4	38.70	3	2.01×10^{-8}
		phiC31	25.84 ± 8.74[a]	9.87 ± 3.14[b]	0.01 ± 0.02[c]	0.01 ± 0.02[c]	3	30.15	3	1.28×10^{-6}
		Bxb1	25.84 ± 8.74[a]	10.47 ± 2.57[b]	0.04 ± 0.10[c]	0.01 ± 0.02[c]	3	30.53	3	1.07×10^{-6}

Different letters indicate significant differences among the data in each line obtained by the Kruskal-Wallis test at the 5% statistical probability level.

Supplementary Table 3. Number of PCR cloned sequences after Int activity on the switch GFP systems and observed covered mutations in HEK 293T, bovine fibroblast and plant protoplast cells.

	Cell type	Sequence description	Int2	Int 4	Int 5	Int 7	Int 9	Int 13	phiC31	Bxb1
Switch GFP system	HEK 293T	Total sequenced clones	7	9	16	16	16	17	10	9
		Selected high quality sequences	11	16	31	31	31	31	20	18
		attL coverage mutations	0	0	0	0	0	0	0	0
		egfp coverage mutations	2 SNPs	3 SNPs	6 SNPs	4 SNPs	7 SNPs<br>2 deletions	0	4 SNPs	5 SNPs
		attR coverage mutations	0	0	0	0	0	0	0	0
	Bovine Fibroblast	Total sequenced clones	11	18	19	24	16	24	10	7
		Selected high quality sequences	16	32	33	48	32	44	20	14
		attL coverage mutations	0	0	0	0	0	0	0	0
		egfp coverage mutations	2 SNPs	5 SNPs	5 SNPs<br>1 deletion	4 SNPs<br>1 deletion	3 SNPs	1 SNP	9 SNPs	3 SNPs<br>1 deletion
		attR coverage mutations	0	1 SNP	0	0	0	0	0	0
	Protoplast	Total sequenced clones	29	37	18	36	12	40	23	15
		Selected high quality sequences	50	66	32	72	23	74	33	28
		attL coverage mutations	0	0	0	0	0	0	0	0
		egfp coverage mutations	2 SNPs<br>1 deletion	2 SNPs	3 SNPs	10 SNPs	0	5 SNPs	4 SNPs	8 SNPs
		attR coverage mutations	0	0	0	1 SNP	0	0	0	0

Supplementary Table 4. Summary of the statistical analysis of the EGFP-positive cell percentages obtained by flow cytometry assays with the switch promoter system in plant protoplasts.

Switch Promoter system - Protoplast	Mean ± sd	pGFP	39.12 ± 12.30[a]
		pIE Int2 + pSP	32.21 ± 5.88[b]
		pIE Int4 + pSP	38.57 ± 10.60[ab]
		pIE Int5 + pSP	12.55 ± 4.57[c]
		pSP	7.35 ± 2.46[d]
		pIE Int2	0.00 ± 0.00[e]
		pIE Int4	0.01 ± 0.02[e]
		pIE Int5	0.01 ± 0.02[e]
	Assays Repetition (n)		3
	Kruskal-Wallis statistics (χ^2)		64.99
	df		7
	p-value		1.51×10^{-11}

Different letters indicate significant differences among the data obtained by the Kruskal-Wallis test at the 5% statistical probability level.

Supplementary Table 5. Number of PCR cloned sequences after Int activity on the switch promoter system and observed covered mutations in protoplasts.

	Int2		Int 4		Int 5	
Switch Promoter system - Protoplast	Total sequenced clones	32	Total sequenced clones	28	Total sequenced clones	37
	Selected high quality sequences	56	Selected high quality sequences	39	Selected high quality sequences	54
	attL Int 2 coverage mutations	0	*attB* Int 2 coverage mutations	0	*attB* Int 2 coverage mutations	0
	attP Int 4 coverage mutations	0	***attL*** Int 4 coverage mutations	0	*attB* Int 4 coverage mutations	2 SNPs
	attP Int 5 coverage mutations	0	*attP* Int 5 coverage mutations	0	***attL*** Int 5 coverage mutations	1 deletion
	5' end CaMV 35S promoter coverage mutations	0	5' end CaMV 35S promoter coverage mutations	0	5' end CaMV 35S promoter coverage mutations	1 SNP
	3' end CaMV 35S promoter coverage mutations	0	3' end CaMV 35S promoter coverage mutations	0	3' end CaMV 35S promoter coverage mutations	0
	attB Int 5 rc coverage mutations	0	*attB* Int 5 rc coverage mutations	0	***attR*** Int 5 coverage mutations	0
	attB Int 4 rc coverage mutations	3 SNPs	***attR*** Int 4 coverage mutations	0	*attP* rc Int 4 coverage mutations	0
	attR Int 2 coverage mutations	0	*attP* rc Int 2 coverage mutations	1 SNP	*attP* rc Int 2 coverage mutations	0
	5' end *egfp* CDS coverage mutations	1 SNP	5' end *egfp* CDS coverage mutations	0	5' end *egfp* CDS coverage mutations	0

rc: reverse complement orientation

Supplementary Table 6. Number of PCR cloned sequences after Int activity on the switch GFP system and observed covered mutations in PBMCs.

	Cell type	Sequence description	Int 4			Int 13			phiC31			Bxb1		
			Donor 1	Donor 2	Donor 3	Donor 1	Donor 2	Donor 3	Donor 1	Donor 2	Donor 3	Donor 1	Donor 2	Donor 3
Switch GFP system	PBMC	Total sequenced clones	10	8	8	11	9	11	9	9	11	9	10	12
		Selected high quality sequences	17	16	14	22	17	20	17	16	19	17	20	22
		attL coverage mutations	0	1 SNP	0	0	1 SNP	0	0	0	0	1 SNP	0	1 SNP
		egfp coverage mutations	3 SNPs	1 SNP	6 SNPs	10 SNPs	5 SNPs	7 SNPs	1 SNP	2 SNPs	3 SNPs	5 SNPs	3 SNPs	0
		attR coverage mutations	0	0	0	0	0	1 SNP	0	0	0	2 SNPs	0	0

Supplementary Table 7. Identification, sequences and target systems of the primers used in this study to amplify *attL* and *attR* sites formed after Int activity in HEK 293T cells, bovine fibroblasts, plant protoplasts and PBMCs.

Switch GFP system	Primers to amplify *attL*		
	Promoter	Forward primer (5’ > 3’)	Target System
	EFa_966F	TTCTCGAGCTTTTGGAGTACGTCGTCTTTAGGTTG	Mammalian
	35S_282F	ATTGATGTGATATCTCCACTGACGTAAGGGATGACGCAC	Plant
	attR	Reverse primer (5’ > 3’)	Target System
	attR _Int2_R	GTGTCTACGCGAGATTCTCGCCGGACCGTCGACATACTGC	General
	attR _Int4_R	AGTTTTCAACCCTTGATTTGAATAAGACTGCTGCTTGTGT	General
	attR _Int5_R	ATAACTCTCCTGGGAGCGCTACACGCTGTGGCTG	General
	attR _Int7_R	CTGTGTGAGAGTTAAGTTTACATGGGCAAAGTTGATGAC	General
	attR _Int9_R	TGGAAGTGTGTATCAGGTAACTGGATACCTCATC	General
	attR _Int13_R	GTAGAACTTGACCAGTTGGTCCTGTAAATATAAGCAATCC	General
	attR _phiC_R2	CCAACTGGGGTAACCTTTGGGCTCC	General
	attR _Bxb1_R2	CTGGTCAACCACCGCGGTCTCCGTCGTCAGGATC	General
	Primers to amplify *attR*		
	attL	Forward primer (5’ > 3’)	Target System
	attL_Int2_F	GGAGTAGCTCTTCGCCCGAGAACTTCTGCAAG	General
	attL_Int4_F	CGACCTGAAATTTGAATTAGCGGTCAAATAATTTGTA	General
	attL_Int5_F	GACGGCCTGGGAGCGTTGACAACTTGCGCACC	General
	attL_Int7_F	GTCCGTCTGGGTCAGTTGCCTAACCTTAACTTTTAC	General
	attL_Int9_F	ATAATTGGCGAACGAGGTATCTGCATAGTTATTCCGAAC	General
	attL_Int13_F	TCCAGATCCAGTTGTTTTAGTAACATAAATACA	General
	attL_phiC_F	TGCCAGGGCGTGCCCTTGAGTTCTCTCAGT	General
	attL_Bxb1_F	TGTCGACGACGGCGGTCTCAGTGGTGTACGGT	General
	Backbone	Reverse primer (5’ > 3’)	Target System
	TermiAni_205R	AATGATTTGCCCTCCCATATGTCCTTCCGAGTG	Mammalian
	NOSt_283R	ATAACAATTTCACACAGGAAACAGCTATGACATGATTACG	Plant
	BB_Termi_R*	GTAAAACGACGGCCAGTGAATTGTAATACGACTC	Plant
Switch Promoter system	Primers to amplify *attL*		
	Backbone and Promoter	(5’ > 3’)	Target System
	Pré_Ints_sitesAt_312F	GCGAAAGGGGGATGTGCT	Plant
	35S_125R	TAGGAGCCACCTTCCTTTTCC	Plant
	Primers to amplify *attR*		
	Promoter and *egfp*	(5’ > 3’)	Target System
	35S_64F	ATCCTTCGCAAGACCCTTCC	Plant
	SGFP_150R	TGGTGCAGATGAACTTCAGG	Plant

*used only with attL_phiC_F and attL_Bxb1_F

Supplementary Methods
Genetic part sequences used to build the vector sets (5’ > 3’)

Integrase recognition sites (orange: core)

attB Int 2
ggacggcgcagaaggggagtagctcttcgccggaccgtcgacatactgctcagctcgtc
attP Int 2 reverse complement sequence
agccaagagcagtgccttgcagaagttctcgggcgagaatctcgcgtagacacatacatgagc

attB Int 4
ttccaaagagcgcccaacgcgacctgaaatttgaataagactgctgcttgtgtaaaggcgatgatt
attP Int 4 reverse complement sequence
aaacgaattacaaattatttgaccgctaattcaaatcaagggttgaaaactttgtaattttt

attB Int 5
gagcgccggatcagggagtggacggcctgggagcgctacacgctgtggctgcggtcggtgc
attP Int 5 reverse complement sequence
cagatcagggtgcgcaagttgtcaacgctcccaggagagttatcgacttgcgtattaggg

attB Int 7
agacgagaaacgttccgtccgtctgggtcagttgggcaaagttgatgaccgggtcgtccgttcctt
attP Int 7 reverse complement sequence
aagctgaacctgcgtaaaagttaaggttaggcatgtaaacttaactctcacacaggtttataacacc

attB Int 9
tttatattgcgaaaaataattggcgaacgaggtaactggatacctcatccgccaattaaaatttg
attP Int 9 reverse complement sequence
taattggaagttcggaataactatgcagatacctgatacacacttccaacaaaaacaaccac

attB Int 13
gcatacattgttgttgtttttccagatccagttggtcctgtaaatataagcaatccatgtgagt
attP Int 13 reverse complement sequence
tattcggagttgtatttatgttactaaaacaactggtcaagttctacaaatacaaccgttattg

attB phiC31
tgccagggcgtgcccttgggctccccgggcgcg
attP phiC31 reverse complement sequence
ccaactgagagaactcaaaggttaccccagttg

attB Bxb1
tcggccggcttgtcgacgacggcggtctccgtcgtcaggatcatccgggc
attP Bxb1 reverse complement sequence
gtcggggtttgtaccgtacaccactgagaccgcggtggttgaccagacaaaccacgac

Expected *attL* and *attR* sites resulting from inversion recombination (orange: core)

attL Int 2
ggacggcgcagaaggggagtagctcttcgcccgagaacttctgcaaggcactgctcttggct
attR Int 2
gacgagctgagcagtatgtcgacggtccggcgagaatctcgcgtagacacatacatgagc

attL Int 4
ttccaaagagcgcccaacgcgacctgaaatttgaattagcggtcaaataatttgtaattcgttt
attR Int 4
aatcatcgcctttacacaagcagcagtcttattcaaatcaagggttgaaaactttgtaatttttt

attL Int 5
gagcgccggatcagggagtggacggcctgggagcgttgacaacttgcgcaccctgatctg
attR Int 5
gcaccgaccgcagccacagcgtgtagcgctcccaggagagttatcgacttgcgtattaggg

attL Int 7
agacgagaaacgttccgtccgtctgggtcagttgcctaaccttaacttttacgcaggttcagctt
attR Int 7
aaggaacggacgacccggtcatcaactttgcccatgtaaacttaactctcacacaggtttataacacc

attL Int 9
tttatattgcgaaaaataattggcgaacgaggtaactgcatagttattccgaacttccaatta
attR Int 9
caaattttaattggcggatgaggtatccagatacctgatacacacttccaacaaaaacaaccac

attL Int 13
gcatacattgttgttgtttttccagatccagttgttttagtaacataaatacaactccgaata
attR Int 13
actcacatggattgcttatatttacaggaccaactggtcaagttctacaaatacaaccgttattg

attL phiC31
tgccagggcgtgcccttgagttctctcagttgg
attR phiC31
cgcgcccggggagcccaaaggttaccccagttg

attL Bxb1
tcggccggcttgtcgacgacggcggtctcagtggtgtacggtacaaaccccgac
attR Bxb1
gcccggatgatcctgacgacggagaccgcggtggttgaccagacaaaccacgac

Mammalian plasmid parts

Ubiquitin C promoter
ggcctccgcgccgggttttggcgcctcccgcgggcgcccccctcctcacggcgagcgctgccacgtcagacgaaggg
cgcagcgagcgtcctgatccttccgcccggacgctcaggacagcggcccgctgctcataagactcggccttagaaccc
cagtatcagcagaaggacattttaggacgggacttgggtgactctagggcactggttttctttccagagagcggaacagg
cgaggaaaagtagtcccttctcggcgattctgcggagggatctccgtggggcggtgaacgccgatgattatataaggac
gcgccgggtgtggcacagctagttccgtcgcagccgggatttgggtcgcggttcttgtttgtggatcgctgtgatcgtcacttg
gtgagtagcgggctgctgggctggccggggctttcgtggccgccgggccgctcggtgggacggaagcgtgtggagag
accgccaagggctgtagtctgggtccgcgagcaaggttgccctgaactgggggttggggggagcgcagcaaaatggc
ggctgttcccgagtcttgaatggaagacgcttgtgaggcgggctgtgaggtcgttgaaacaaggtgggggggcatggtgg
gcggcaagaacccaaggtcttgaggccttcgctaatgcgggaaagctcttattcgggtgagatgggctggggcaccatc
tggggaccctgacgtgaagtttgtcactgactggagaactcggtttgtcgtctgttgcgggggcggcagttatggcggtgcc
gttgggcagtgcacccgtacctttgggagcgcgcgccctcgtcgtgtcgtgacgtcacccgttctgttggcttataatgcag
ggtggggccacctgccggtaggtgtgcggtaggcttttctccgtcgcaggacgcagggttcgggcctagggtaggctctc
ctgaatcgacaggcgccggacctctggtgaggggagggataagtgaggcgtcagtttctttggtcggttttatgtacctatct
tcttaagtagctgaagctccggttttgaactatgcgctcggggttggcgagtgtgttttgtgaagttttttaggcaccttttgaaat
gtaatcatttgggtcaatatgtaattttcagtgttagactagtaaattgtccgctaaattctggccgtttttggctttttgttagac

EF1alpha promoter
gctccggtgcccgtcagtgggcagagcgcacatcgcccacagtccccgagaagttggggggaggggtcggcaattga
accggtgcctagagaaggtggcgcggggtaaactgggaaagtgatgtcgtgtactggctccgcctttttcccgagggtgg
gggagaaccgtatataagtgcagtagtcgccgtgaacgttctttttcgcaacgggtttgccgccagaacacaggtaagtg
ccgtgtgtggttcccgcgggcctggcctctttacgggttatggcccttgcgtgccttgaattacttccacgcccctggctgcag
tacgtgattcttgatcccgagcttcgggttggaagtgggtgggagagttcgaggccttgcgcttaaggagccccttcgcctc
gtgcttgagttgaggcctggcctgggcgctggggccgccgcgtgcgaatctggtggcaccttcgcgcctgtctcgctgcttt
cgataagtctctagccatttaaaatttttgatgacctgctgcgacgctttttttctggcaagatagtcttgtaaatgcgggccaa
gatctgcacactggtatttcggtttttggggccgcgggcggcgacggggcccgtgcgtcccagcgcacatgttcggcgag
gcggggcctgcgagcgcggccaccgagaatcggacgggggtagtctcaagctggccggcctgctctggtgcctggcct
cgcgccgccgtgtatcgccccgccctgggcggcaaggctggcccggtcggcaccagttgcgtgagcggaaagatggc
cgcttcccggccctgctgcagggagctcaaaatggaggacgcggcgctcgggagagcgggcgggtgagtcacccac
acaaaggaaaagggcctttccgtcctcagccgtcgcttcatgtgactccacggagtaccgggcgccgtccaggcacctc
gattagttctcgagcttttggagtacgtcgtctttaggttggggggaggggttttatgcgatggagtttccccacactgagtgg
gtggagactgaagttaggccagcttggcacttgatgtaattctccttggaatttgccctttttgagtttggatcttggttcattctca
agcctcagacagtggttcaaagtttttttcttccatttcaggtgtcgtga

egfp reverse complement sequence
ttacttgtacagctcgtccatgccgagagtgatcccggcggcggtcacgaactccagcaggaccatgtgatcgcgcttctc
gttggggtctttgctcagggcggactgggtgctcaggtagtggttgtcgggcagcagcacggggccgtcgccgatgggg
gtgttctgctggtagtggtcggcgagctgcacgctgccgtcctcgatgttgtggcggatcttgaagttcaccttgatgccgttct
tctgcttgtcggccatgatatagacgttgtggctgttgtagttgtactccagcttgtgccccaggatgttgccgtcctccttgaag
tcgatgcccttcagctcgatgcggttcaccagggtgtcgccctcgaacttcacctcggcgcgggtcttgtagttgccgtcgtc
cttgaagaagatggtgcgctcctggacgtagccttcgggcatggcggacttgaagaagtcgtgctgcttcatgtggtcggg
gtagcggctgaagcactgcacgccgtaggtcagggtggtcacgagggtgggccagggcacgggcagcttgccggtg
gtgcagatgaacttcagggtcagcttgccgtaggtggcatcgccctcgccctcgccggacacgctgaacttgtggccgttt
acgtcgccgtccagctcgaccaggatgggcaccaccccggtgaacagctcctcgcccttgctcaccat

β-globin poly(A) signal terminator
agcggccgcactcctcaggtgcaggctgcctatcagaaggtggtggctggtgtggccaatgccctggctcacaaatacc
actgagatctttttccctctgccaaaaattatggggacatcatgaagccccttgagcatctgacttctggctaataaaggaa
atttattttcattgcaatagtgtgttggaattttttgtgtctctcactcggaaggacatatgggagggcaaatcatttaaaacatc
agaatgagtatttggtttagagtttggcaacatatgccatatgctggctgccatgaacaaaggtggctataaagaggtcatc
agtatatgaaacagccccctgctgtccattccttattccatagaaaagccttgacttgaggttagattttttttatattttgttttgtgt
tattttttttctttaacatccctaaaattttccttacatgttttactagccagatttttcctcctctcctgactactcccagtcatagctgtc
cctcttctcttatgaagatccctcgacctgcagcccaagcttggcgtaatcatg

Integrase 2 *H. sapiens* codon optimized sequence
atgcccatcgccccggagttcctgagtttggcctatccgggacaagagtttcctgcttatctctacgggagggcttccaggg
atccgaagagaaaaggtcgaagcgtccaaagccaattggacgaagggagggcaacctgtcttgatgctggatggcct
attgctggggaatttaaagatgtagacagatcagcatctgcctatgctaggaggacgcgagatgaatttgaagagatgat
cgcaggaatccaggcgggggagtgccggattcttgtggctttcgaagcgtctcgatattacagagatttggaggcctatgtt
cggcttcggcgagtctgccgggaagcaggcgttcttctttgctataacggtcaggtgtacgatcttagtaagagtgcagata
gaaaggcgacggctcaggatgcggtgaacgcagaaggcgaagccgacgatataagagaaagaaacctccgcacc
accagacttaatgccaagcgagggggagctcatgggcctgtcccagatggatataagagacgatatgatccagattcc
ggagatcttgtcgaccaaatcccgcaccccgacagagccggtttgataactgagatttttaggcgggcagcggcggccg
agccactggccgctatctgccgagatctcaatgagaggggcgaaactacccaccgaggcaaagcatggcagaggca
ccacctgcatgcgatccttcgcaatccagcttatatcggtcatagaagacatctcggtgttgacacaggtaaaggaatgtg
ggcacctatttgcgatgatgaggactttgcggaaacctttcaggctgtccaggaaatcctcagtcttccagggagacagtt
gagcccgggccctgaagcgcaacatttgcagacagggatcgcactttgcggagaacacccggacgaaccgccgctta
ggagtgtgactgttcgaggccgcacaaattacaattgttccacaagatacgatgtggcgatgcgagaagaccgcatgga
tgcgttcgtcgaggagtccgtcatcacgtggctggcctccgacgaagcagttgcagcttttgaagataatactgacgatga
acggacaagaaaagcgaggatcagactcaaagttcttgaagagcagctcgaggctgcacaaaaacaggctagaac

actccgcccggacggaatgggcatgctccttagtatagattccttggccggcttggaagccgaacttactccacagatag
acaaggcgaggcaggagagtcggtctttgcacgtacccgctctcttgagagacctgttgggcaaaccgcgagctgatgtt
gaccgcgcatggaacgaagcactcacgttgccccagcgccgaatgatactgcgcatggttgtcactatccgcctcttcaa
agcaggatctcgaggtgtacgggcgatagagccaggtcgaattaccttgagttatgttggagaaccaggatttaagcccg
tgggcggaaatcgagcaaaacagtaa

Integrase 4 *H. sapiens* codon optimized sequence
atgatcaccacacgcaaggtggccatatacgtccgcgtatctacgactaatcaagccgaagagggatattctatacagg
gccaaatcgattcccttatcaagtactgcgaggctatgggttggatcatatatgaggaatatactgacgcagggttttcagg
cggcaaaattgatcggcccgccatgagtaagcttattactgatgccaaacacaagagattcgataccattctggtgtaca
aattggatcggctgtcaagaagcgtgcgggatacactttacttggttaaggatgtatttaatcaaaacaacatccacttcgtc
tcccttcaggagaatatcgacacatcttccgcgatgggaaatctttttttgaccctcctttctgccatagcggagtttgagcga
gagcagatcacggagcgcatgaccatggggaaaataggtcgggccaaatcagggaagacaatggcgtggacctata
caccgttcggctacgattataataaggagaagggtgaacttatacttgatcctgccaaggcacccatcgtgaaaatgatat
acaccgattacttgaagggtatgtcaattcaaaagatagtggataaactcaataaaatggattacaacggcaaggattgc
acatggtttccccacggcgtgaaacatctcttggataatccggtgtactacggtatgacaagatataacaataagcttttccc
cggcaatcatcagccgataattacaaaggagttgtttgataagacccagcgcgagagacaacggaggcgactcggca
tcgaggaaaaccattatacaataccgtttcaggcaaaatatatgctcagtaagttcctcaggtgtcgccagtgcggctccc
gaatggggcttgaattgggtcgcccgcgcaagaaggaaggcaagcgcagtaagaaatactactgcctcaatagccga
ccgaaacggacagcttcatgtgacactcccctgtatgacgccgaaaccctggaagattatgtgctgcacgaaatagcta
aaatacagaaagacccctcaatagcaagtagacagaagcatattgaagatcacgaactcaaatataagcgagaacg
catagaggcaaatataaataaaaccgtgaatcaacttagcaagctgaacaacctttaccttaacgacctcattactcttga
ggacctcaagacacagactaacacactcatcgcgaaaaagcggctgttggagaatgaacttgacaagacatgcgata
atgacgacgaacttgacagacaagagacaatagcggactttctggcattgccagacgtttggactatggactatgaaggt
caaaagtatgccgtggagcttctcgttcagcgcgtcaaagttgacagagacaacatagacatacattggacgttttaa

Integrase 5 *H. sapiens* codon optimized sequence
atgccgggaatgaccacggaaaccggaccagatccggcaggtttgatagacctgttttgccgcaaatctaaggcagtg
aaaagccgggctaatggagctgggcaacggagaaaacaggaaatctctattgctgctcaggagacgctggggcgca
aggtcgctgccctgctcggcatgcaagtccgccatgtgtggaaggaagtaggtagcgcgagtcgattcagaaagggaa
aggcaagggatgatcagagtaaagcccttaaagcacttgagagtggggaggtcggggcgctctggtgctatcggcttg
accgatgggataggggaggcgcgggagcaattcttaaaattatagagcccgaggatggcatgccgcgaagattgctttt
cggctgggatgaggatacaggtcgacccgtattggattctacaaataaaagggaccggggtgagctgatcagacgagc
ggaggaagcccgagaagaagcagaaaaactgtcagagcgcgtccgcgatactaaggcacatcagcgcgagaacg
gggaatgggtaaacgcgcgcgcaccatatggcctccgagtggtcttggtgactgttagtgatgaggagggcgacgagta
tgatgaaaggaaattggctgcagacgatgaagatgctggtggacccgatggtctcacaaaagctgaagcggcacggct
ggtcttcactctcccggttaccgaccgactttcctacgcaggtaccgcccatgcaatgaacactcgggaaattccatcacc
gacaggcggtccatggatagctgtgacagttagggacatgatccagaaccccgcttatgcggggtggcagaccacag
gcaggcaggatggaaaacaaaggcgcttgacattctacaacggagaggggaaaagggtctctgtcatgcacggccc
accccctggtaacagatgaagaacaggaagcggccaaggcggcagtcaaaggcgaagacggagtaggtgtgccgct
tgacggctcagatcatgacacacgccgcaaacatctcctcagtggtaggatgcgatgtccgggatgtggtggctcatgct
cctactcaggtaacggttaccggtgttggagatctagtgttaaaggggggctgtccagcaccgacgtacgtggccagaaa
gtcagtcgaggagtatgtggcttttaggtgggccgctaaactggcggcctccgagcctgatgacccctttgtaatagctgtg
gcagatcgctgggcagctttgacacacccacaggcttccgaagatgaaaaatacgctaaagctgcggttcgagaagca
gagaagaatcttggccggctgctgcgagaccggcagaacggcgtctatgatgggcccgcggagcagttctttgcgccg
gcttatcaagaagccctttcaacgctgcaggcggctaaggatgctgtctcagaaagctccgcatccgcagcagtggatgt
aagttggatagtggatagttccgattacgaggagctgtggcttcgggcgaccccgacaatgaggaatgcgattattgata
cttgcatcgacgagatttgggtggcaaagggacaacgagggagaccgtttgatggagatgaacgggtcaagattaaat
gggcagctcggacgtaa

Integrase 7 *H. sapiens* codon optimized sequence
atgaaggtagctatatacgtccgcgtctcaacagatgaacaagctaaggagggattctccattccggcgcagagagag
aggctcagggctttttgtgccagtcaagggtgggagatcgtgcaagaatatatcgaagagggctggagcgcaaaggac
cttgatcgcccacagatgcagcgattgctgaaggacataaaaaaagggaacatagacatagtgctggtctataggcttg
accgacttaccaggtcagtcctcgatctctacctgttgctccaaacattcgaaaagtataacgtagccttccgatccgctac

cgaggtttacgacacaagcacagctatggggcgactttttattaccttggttgccgctttggcgcaatgggaacgcgaaaa
tctcgcagagagagtaaagttcggcatagaacagatgatcgatgaaggcaaaaagcctggtgggcattcaccatatgg
ctataagttcgataaagacttcaactgtacgattattgaagaggaggcagacgttgttaggatgatataccgcatgtactgc
gatggatatggctataggagtattgccgatcgactgaatgagcttatggtaaaaccaagaatagctaaagagtggaatca
taacagtgttcgcgacatccttactaacgacatatatatcggcacgtaccgatggggagacaaggtggttccaaacaacc
acccacctatcattagtgaaactttgtttaagaaagcgcaaaaagaaaaagaaaagcgaggagtggatcgaaagaga
gttggtaagttcctgtttactggtcttctccagtgttgtaattgtggaggacacaagatgcaggggcacttcgacaaacgcga
gcaaaagacttactacagatgcacgaagtgtcacaggattaccaatgagaaaaatatcctggaacccttgttggatgag
atacagttgctgataacatccaaggaatattttatgtctaaatttagtgaccgatatgatcaacaggaagtagtagacgtttct
gctcttacgaaagaactggagaagatcaagcgccaaaaggagaagtggtatgatctctatatggacgatcgcaatccc
attcctaaggaagagttgtttgcgaaaatcaatgaactgaacaagaaagaagaagagatatattccaagttgagcgag
gtagaggaggacaaagagcccgtcgaagagaaatgcaatagactgtccaagatgattgattttaagcagcaattcgaa
caggcgaacgacttcacgaaaaaggaattgttgtttagcatatttgaaaaaatcgtaatttatagggaaaaaggtaagct
gaagaagataactctggactacactctcaagtaa

Integrase 9 *H. sapiens* codon optimized sequence
atgaaggtggccatttacacgcgagtgagtactttggaacagaaggagaaggggcatagtattgaggaacaggaacg
caagctgcgggcctacagtgacatcaacgattggaaaatccataaagtatacactgacgcaggatacagcggggcga
aaaaggatcgcccagcacttcaggaaatgttgaatgagattgataattttgacctcgtcctcgtctacaagttggataggctt
acaaggagtgtgaaagacctccttgagattctcgagctgtttgaaaacaagaacgtcctgtttagatcagccactgaggtc
tacgatactacgtcagctatgggtcggctctttgttactctggttggcgctatggctgagtgggaacgcacgacaattcagg
agagaactgcgatggggcgacgagcgagtgctcggaaaggacttgccaagactgttccaccgttttactacgacaggg
tgaatgataagtttgttcccaacgaatataaaaaggtcctgcgcttcgcggtcgaagaggcaaaaaaagggacaagctt
gcgagagatcaccattaaactgaataactccaagtataaagctccattgggcaagaattggcatcgaagtgtgatagga
aatgcactgacgagcccggttgccaggggggcacttggtctttggcgacatttttgtcgagaacacccatgaggcgattata
agtgaagaagagtacgaggagataaaactcaggatctctgagaaaacgaatagcacaatagttaagcacaacgcca
tattcagatcaaaactgctgtgtcccaattgcaaccagaagctgacactgaataccgtgaaacataccccaaagaacaa
ggaggtttggtattctaaactgtacttctgcagtaattgcaagaacacaaagaacaaaaatgcatgcaacattgacgagg
gagaggtgctgaagcaattttataattacctgaagcaatttgacctgacgtcctacaaaatagaaaaccagccgaaaga
aattgaggatgtggggatcgacatcgaaaagcttcgcaaggagcgggcacgatgccagacacttttcattgagggcatg
atggacaaagatgaagcttttcccatcatttcaagaatagataaggagatccatgagtatgagaagcggaaggataacg
acaaggggaagacgttcaactacgagaagataaagaatttcaagtactcccttcttaatgggtgggaactcatggaaga
cgagctgaaaacggaattcatcaaaatggctatcaaaaacatccacttcgagtatgtgaaggggattaaaggtaaaag
acaaaatagtctcaagataaccggtatagagttttactaa

Integrase 13 *H. sapiens* codon optimized sequence
atggcagtcggaatttatattcgagttagtacccaagaacaggcgtccgaaggccactctatagaatcccaaaaaaaga
agctggccagctactgcgagattcagggatgggacgattatcgcttctacatcgaggaaggtatctccgggaaaaacac
aaatcggccaaagttgaaactgctcatggaacatattgaaaagggaaagattaatattctcctcgtatatcgcctcgaccg
actcacacgctctgtgatcgatttgcacaagctgcttaactttttgcaggagcatgggtgtgcatttaaatcagcgacagag
acctatgatacgacgacagcgaatggtcgcatgtccatgggtattgtcagtctcttggcccaatgggaaacagagaatat
gtccgaacggataaaacttaaccttgaacacaaggtgctcgtcgaaggggagagagtcggagcgatcccctacggatt
cgacctctccgacgacgaaaagcttgttaagaacgaaaaatctgccatattgctggacatggtggaaagagtcgagaa
cgggtggtcagtcaatcggattgtcaactatttgaatcttactaataatgatcggaactggtccccgaacggtgttctgcgcc
tgctgcggaacccggccctgtacggcgccacacggtggaacgacaagatcgctgaaaacacgcacgaaggcattat
ctctaaagaaagatttaatcggcttcagcagatattggcagaccgctccatacaccaccgaagggacgtgaaaggtact
tatatatttcaaggggtcttgcgatgtcccgtctgcgaccaaaccctgagtgtaaaccgctttattaaaaaacggaaggatg
ggacggagtattgcggcgtcctgtatagatgccaaccatgcataaaacagaataaatacaatcttgccatcggagaagc
tcgattcctgaaggctctcaatgaatatatgtccacagtagagtttcagacggttgaggatgaagtaatacccaaaaaaa
gtgagcgagaaatgctcgagtcacagcttcaacagatcgctagaaagcgagagaagtaccaaaaggcgtgggcctc
agaccttatgagtgacgatgagtttgaaaagctgatggtagaaacgagagaaacgtatgatgagtgtaaacaaaagctt
gagtcttgtgaagacccaataaagatcgacgagacttaccttaaagagatagtttatatgttccaccagacgttcaacgat
ctggagtctgagaagcaaaaagaatttatctcaaagtttattcgcacgattcgatacactgtaaaggagcaacaaccaatt
cggccggataaatctaagacggggaaagggaagcagaaagttatcataacggaggttgagttctatcagtaa

phiC31 *H. sapiens* codon optimized sequence
atggacacatacgcaggagcgtatgatcggcagtcccgcgagcgagagaacagcagtgccgcaagcccggcgacc
cagcgatccgccaatgaagataaagcggcagacctccagcgggaagtagaaagagatggaggtaggtttagatttgt
aggacatttttccgaggctccaggaacctccgcattcggcacggcagaacgacctgagtttgagcgcatcctgaatgaat
gtagggccggcaggctcaacatgatcatagtatatgacgtgtcacgcttctccaggctcaaggtgatggacgctattccaa
tcgtctcagagcttctcgctctcggagtgaccatcgtcagcacccaagaaggtgtcttcaggcaggggaacgtaatggat
cttatacatcttattatgcgcctggacgcttcccacaaggaatcatccctcaagtccgcgaagatactcgatactaagaacc
tgcagcgcgagttgggtggttatgttggtggaaaggccccatacggtttcgaattggtgagcgaaacgaaggagattacc
aggaatgggcgcatggttaatgttgtaataaacaaactggctcacagcacaaccccactgaccggccccttcgagtttga
acccgacgtgattagatggtggtggcgggagatcaagacgcataagcaccttccgtttaagccggggtcacaggccgc
aattcatcctggttctatcacaggtttgtgcaaacggatggatgctgatgcggtgcctacccgaggggaaacgatcggca
aaaagacggcttcatcagcctgggatccggccacggttatgcgcatacttagagacccacgcatcgcgggctttgcagc
cgaggttatatataaaaaaaagccagatggcacacctaccaccaaaatagaagggtaccggattcagcgagatccga
ttactctcaggcctgttgagctggattgcggtccgataattgagcccgctgaatggtatgaactccaggcgtggctcgacgg
tagggggcgcgggaagggcctgagccgaggccaggctatactgtccgccatggataaactgtattgcgagtgtggtgc
ggtaatgacctccaaaagaggagaagaaagcatcaaggattcatatcgatgcagaaggcgcaaagttgtggacccct
ctgcacctggtcaacatgaggggacatgcaatgtatcaatggctgcgctcgacaagtttgtagccgagcggatattcaat
aagattagacacgccgagggagacgaggagactcttgcactgctctgggaggcagcccggcgctttgggaaactgac
ggaggcccctgaaaaaagtggtgaacgcgctaaccttgtagcggagagagcggatgcactcaacgcgttggaagaa
ctttatgaggaccgggcagcaggcgcctatgacggtccagtcgggcgaaagcactttaggaaacaacaggccgcactt
actcttcgccagcaggggggcggaggaacgactcgctgagttggaagcggcagaggcccccaagttgcccttggacca
gtggttccccgaagatgccgatgcagatccaacagggcctaagagctggtgggggcgggcgtccgtcgatgataaga
gagtgtttgttggcttgttcgtggacaagattgtggtaaccaagtcaactactggtcggggtcaaggtaccccaatagaga
agcgggccagcataacatgggcaaagcccccgacggacgacgatgaggacgatgctcaagacggaaccgaagat
gttgctgcttag

Bxb1 *H. sapiens* codon optimized sequence
atgcgggcactggtggtaattaggctctccagagttactgacgctacgaccagccctgagaggcaacttgaaagctgcc
agcagctctgtgcgcaacggggctgggacgtcgttggagtggcggaagacctcgatgtttccggtgcagtagatcccttc
gataggaagcggcgcccgaatttggctcgatggctcgctttcgaagagcaaccattcgatgtgatcgttgcctatcgagtc
gacaggttgacaagaagtattcgccatcttcaacaattggttcattgggcagaagatcacaaaaaattggttgtctccgcta
cggaagcacactttgatacaactacaccgtttgcagcagttgtcatagctctgatgggtaccgttgcacaaatggaattgg
aagctattaaagagcgaaatcggagcgccgcgcactttaacatcagggcaggcaagtaccgcggcagtctcccaccc
tggggctacctgccgactagagtggatggcgaatggcggttggtgcccgacccggtccaacgggaaagaatccttgaa
gtgtaccatcgagttgtggacaatcacgagccccttcacctggtagctcatgatttgaatcgccggggagttttgagcccga
aagattatttcgcccagctgcaagggagggaaccacagggtcgggagtggagtgctactgctctgaaaagatccatgat
tagcgaagccatgctgggatacgcaacccttaacggcaaaaccgttagggacgatgacggagcgcccctggttcggg
ccgaaccaattcttactcgcgagcaactggaagcactgcgggctgagctggtcaaaacatcacgggctaaaccggcg
gtgagcacaccaagtttgttgcttcgagtattgttttgcgccgtttgtggagagccagcatacaagtttgccgggggtgggag
aaaacatccccgctatagatgccgcagcatgggcttcccgaagcattgtggaaacggtacagtagcgatggctgagtg
ggatgcattctgcgaagaacaagtccttgacttgcttggcgacgcagaacgcctggaaaaggtatgggtagctgggtcc
gactccgctgtagaacttgctgaagtcaatgcagaactggtcgatctcacatcccttattggctctccagcgtaccgagctg
gctccccgcaacgcgaggcacttgatgccagaatagcggcactcgccgcgagacaagaggaattggaggggttgga
agctagaccttctggatgggaatggagggaaacaggccagcggttcggggattggtggcgcgagcaagatactgcgg
cgaagaatacgtggcttaggagcatgaacgtccggctgacgttcgacgttagagggggcttaccaggactatcgatttc
ggagatttgcaggagtatgaacaacacttgaggcttggtagcgtagtggagagactgcatacagggatgtcttag

Plant plasmid parts

Actin2 promoter
aaaatttagaacgaacttaattatgatctcaaatacattgatacatatctcatctagatctaggttatcattatgtaagaaagttt
tgacgaatatggcacgacaaaatggctagactcgatgtaattggtatctcaactcaacattatacttataccaaacattagtt
agacaaaatttaaacaactattttttatgtatgcaagagtcagcatatgtataattgattcagaatcgttttgacgagttcggat
gtagtagtagccattatttaatgtacatactaatcgtgaatagtgaatatgatgaaacattgtatcttattgtataaatatccata

aacacatcatgaaagacactttctttcacggtctgaattaattatgatacaattctaatagaaaacgaattaaattacgttga
attgtatgaaatctaattgaacaagccaaccacgacgacgactaacgttgcctggattgactcggtttaagttaaccacta
aaaaaacggagctgtcatgtaacacgcggatcgagcaggtcacagtcatgaagccatcaaagcaaaagaactaatc
caagggctgagatgattaattagtttaaaaattagttaacacgagggaaaaggctgtctgacagccaggtcacgttatcttt
acctgtggtcgaaatgattcgtgtctgtcgattttaattatttttttgaaaggccgaaaataaagttgtaagagataaacccgc
ctatataaattcatatattttcctctccgctttgaattgtctcgttgtcctcctcactttcatcagccgttttgaatctccggcgacttg
acagagaagaacaaggaagaagactaagagagaaagtaagagataatccaggagattcattctccgttttgaatcttc
ctcaatctcatcttcttccgctctttctttccaaggtaataggaactttctggatctactttatttgctggatctcgatcttgttttctca
atttccttgagatctggaattcgtttaatttggatctgtgaacctccactaaatcttttggttttactagaatcgatctaagttgacc
gatcagttagctcgattatagctaccagaatttggcttgaccttgatggagagatccatgttcatgttacctgggaaatgattt
gtatatgtgaattgaaatctgaactgttgaagttagattgaatctgaacactgtcaatgttagattgaatctgaacactgtttaa
ggttagatgaagtttgtgtatagattcttcgaaactttaggatttgtagtgtcgtacgttgaacagaaagctatttctgattcaatc
agggtttatttgactgtattgaactctttttgtgtgtttgcagctcataaaaa

CaMV 35S promoter

taattcatcaaattataactatatagaccctaatttcatatgagacttttcaacaaagggtaatatccggaaacctcctcggat
tccattgcccagctatctgtcactttattgtgaagatagtggaaaaggaaggtggctcctacaaatgccatcattgcgataa
aggaaaggccatcgttgaagatgcctctgccgacagtggtcccaaagatggacccccacccacgaggagcatcgtgg
aaaaagaagacgttccaaccacgtcttcaaagcaagtggattgatgtgatatctccactgacgtaagggatgacgcaca
atcccactatccttcgcaagacccttcctctatataaggaagttcatttcatttggagaggactaactgtgccaaattatcttcc
aaactcctaatccaatt

egfp reverse complement sequence

ttacttgtacagctcgtccatgccgagagtgatcccggcggcggtcacgaactccagcaggaccatgtgatcgcgcttctc
gttggggtctttgctcagggcggactgggtgctcaggtagtggttgtcgggcagcagcacggggccgtcgccgatgggg
gtgttctgctggtagtggtcggcgagctgcacgctgccgtcctcgatgttgtggcggatcttgaagttcaccttgatgccgttct
tctgcttgtcggccatgatatagacgttgtggctgttgtagttgtactccagcttgtgccccaggatgttgccgtcctccttgaag
tcgatgcccttcagctcgatgcggttcaccagggtgtcgccctcgaacttcacctcggcgcgggtcttgtagttgccgtcgtc
cttgaagaagatggtgcgctcctggacgtagccttcgggcatggcggacttgaagaagtcgtgctgcttcatgtggtcggg
gtagcggctgaagcactgcacgccgtaggtgaaggtggtcacgagggtgggccagggcacgggcagcttgccggtg
gtgcagatgaacttcagggtcagcttgccgtaggtggcatcgccctcgccctcgccggacacgctgaacttgtggccgttt
acgtcgccgtccagctcgaccaggatgggcaccaccccggtgaacagctcctcgcccttgctcaccat

NOS terminator

gaatttccccgatcgttcaaacatttggcaataaagtttcttaagattgaatcctgttgccggtcttgcgatgattatcatataatt
tctgttgaattacgttaagcatgtaatatttaacatgtaatgcatgacgttatttatgagatgggtttttatgattagagtcccgca
attatacatttaatacgcgatagaaaacaaaatatcgcgcgcaaacttggataaattatcgcgcgcggtgtcatctatgag
gactagatcg

Integrase 2 *A. thaliana* codon optimized sequence

atgccaatagcgccagaatttctctcacttgcttatccaggtcaagagttccccgcgtacctttacggacgagcttctaggg
atccgaaacgtaagggaagatctgttcaatctcaattagacgaagggagagctacgtgtctcgatgcagggtggccaatt
gctggtgagttcaaagatgtcgatagatcggcctccgcatatgctagacgtacacgtgacgaatttgaggaaatgatagc
aggaattcaggccggcgaatgtagaattctcgtggcctttgaagcatccagatattatcgagatttagaggcgtatgtcag
gcttagacgagtatgtcgtgaagccggagttctgttgtgttacaacggtcaggtttacgatttaagcaaatccgccgaccga
aaagcgaccgcacaggacgccgttaatgcagagggtgaggcagatgatattagagaacgtaatttgagaacaactag
attgaatgcaaagagaggaggcgctcatggccccgtgcctgatgggtataaaaggagatacgaccctgattcaggtgat
ttagttgatcaaatcccacatccagatagagctggtctgataacagaaatattccgtagggcagcagcggccgagcctct
cgctgctatatgtcgagacttaaacgagagaggagaaacaactcatagaggtaaagcatggcaaaggcaccatctgc
acgctatcctgagaaatccagcgtacatcggtcataggagacatcttggcgtggatacaggtaaaggaatgtgggcacc
aatatgtgatgatgaagacttcgctgaaacattccaagctgtccaggaaatactctctttaccaggaaggcaactatcgcc
cggaccagaagcccaacacctacagactggcattgctttatgcggtgagcatccagacgagccaccactacgttcggtc
actgtaagaggtagaactaattacaactgttctacacgatatgatgttgcgatgagggaggatcgtatggatgcttttgtgga
agaatcagttattacttggcttgcgagtgatgaagcggtggctgctttcgaagataatacagatgatgagagaacgagaa

aagccagaattcgacttaaagttctagaagagcaattagaagccgctcaaaaacaggctagaacactccgtcctgatg
ggatggggatgctcttgtctatcgactctcttgctggtctcgaggccgaacttacgccacagatagacaaggcaaggcaa
gagagcagatccctgcatgtacccgcacttttgcgtgaccttctaggcaagccacgagcagacgttgatcgtgcatggaa
cgaagctctaaccctccctcagagacgtatgatacttaggatggttgttacgattagactattcaaggcgggttctcgtggtg
tcagggcaatagagcctggtcgaattacgctcagctacgttggagaacctggatttaagccagtgggtggtaacagagc
aaaacagtga

Integrase 4 *A. thaliana* codon optimized sequence
atgattacgaccagaaaggtggctatatacgttcgtgtcagcactactaatcaggctgaggaagggtatagtattcaaggt
caaatcgattcactaataaagtactgcgaagctatgggctggatcatctatgaggaatatactgatgccgggttcagtgga
gggaagattgataggcccgctatgagtaaattaatcacggatgcaaagcacaagagatttgatacgattcttgtctataaa
cttgacagattaagtagatccgtcagggacacgttatacctggtgaaggatgtcttcaatcagaacaatatacacttcgtga
gcctacaggaaaacatcgatacttcctcagcgatgggtaatctattcttgaccctcttatcagcgatagctgagtttgagcga
gaacaaattacggagcgaatgacgatgggcaagattgggcgagccaaatctggtaagacaatggcttggacatatact
cctttcggatacgactacaacaaggagaagggagagctcatccttgacccagccaaagctcctatcgtgaagatgattta
caccgactatttaaaagggatgtccatacagaaaattgttgacaagctgaataagatggattacaatggtaaagattgca
cttggttcccgcatggcgtgaagcacctcctagacaatccagtctactatggaatgacaaggtataacaacaaattattcc
ctggaaatcatcagcctattatcaccaaggaactattcgacaagacccagagagagagacagaggcgacgattgggc
attgaagagaatcattacacaattccattccaggctaagtacatgctgtctaaattcttgcgatgcaggcagtgcggttcaa
ggatggggctcgaacttgggagacctcgtaagaaggaaggtaaaaggtcaaagaaatactattgtctgaattctagacc
taagaggacggcttcctgcgacactcccctatacgatgctgagacactggaagattacgtgctgcatgaaattgccaaaa
tccaaaaggatccgtcaatcgcgagtcgacaaaaacatatcgaggatcacgagttaaagtacaagcgtgaacgtattg
aagccaatataaataaaaccgtaaatcaactgtccaagttgaacaatttgtacctaaacgacttgatcaccttggaggac
ctcaagactcagacgaacacacttatagcgaagaaacgtcttctcgaaaacgaacttgataagacatgcgacaacgat
gacgaacttgataggcaggaaactattgccgacttcctggctctacctgatgtgtggacgatggactatgagggccagaa
gtatgctgttgagttgttagtccagagagttaaagtggacagggataacatagatatacattggaccttttaa

Integrase 5 *A. thaliana* codon optimized sequence
atgccaggcatgacgacagagaccggacccgacccggcagggttaatagacctgttctgtagaaaaagcaaagctgt
caaatctagggctaatggggcgggtcaaaggagaaaacaggaaatttccatagcagcgcaggagacgctcgggag
gaaggtagccgcgttgttaggaatgcaagtcaggcatgtatggaaggaggtaggatctgccagcagattcagaaaggg
aaaggcgagggatgaccagtccaaagctctaaaggcccttgagtctggagaggtgggcgcactgtggtgctatcgtcta
gatagatgggatagagggggtgcaggtgctatccttaagataatcgaaccggaggacggtatgcctcgtcgattgttgttt
ggctgggacgaagatacaggcaggccggtgctggacagtactaacaaacgagaccgtggtgaactaatacgacgag
cagaagaggctcgtgaggaggcggaaaagcttagtgagagggtaagagatactaaagcgcatcaaagagaaaacg
gggaatgggtaaatgctagggcgccgtacggactcagggttgtacttgtcacagtatccgatgaagaaggcgatgagta
cgatgaacgaaagcttgcagcagacgatgaagacgctggaggcccggatggtctaacgaaagcggaggccgcacg
actcgtgttcacacttcccgtcactgatcgactatcctacgcgggaacggctcatgctatgaatacccgagaaataccctct
ccgactgggggccatggattgccgtaacggtcagggatatgatacagaaccccgcctacgcgggctggcagacaac
cgggcgacaggacggtaaacaacgaagacttaccttttataatggtgaaggaaagcgtgttagcgtgatgcacggacc
accacttgttacggatgaggaacaggaggcagcgaaggcagcggttaagggagaggatggggtcggagttcccttag
acggttcagaccatgacacgaggcgaaagcatctactctctggccgaatgcgatgtcctggctgcgggggctcctgttca
tacagtggcaatggctatagatgttggagatcttcagtcaaaggcggttgccccgcccccacgtatgtcgcgaggaagtc
cgtagaagagtatgttgcttttaggtgggctgctaagctcgctgcctcagagccggatgatccttttgttatagcggttgcaga
taggtgggcggcgttaacccatcctcaggcgagcgaagatgagaaatatgccaaagctgccgttcgagaggcagaga
aaaatctaggccgtttgctgcgtgatcgacaaaatggagtctacgacgggccggcggagcaatttttcgcacccgcatat
caagaggcattaagcacacttcaggcagccaaggatgccgttagtgaaagctcagcgagtgctgctgtagacgtgagc
tggatcgtagacagtagcgattacgaggagctatggttaagagctacgccgacaatgaggaatgccataatagacacg
tgtatagatgagatttgggttgcgaagggtcagaggggaaggccgttcgatggggacgagcgagtaaagattaaatgg
gcagcgcgtacataa

Integrase 7 *A. thaliana* codon optimized sequence
atgaaggtagcgatctatgtccgtgtgtcaactgatgagcaggccaaggaaggattttccatcccagcgcagagagaaa
gattaagggcattttgtgcctcacaaggctgggagatcgtacaggagtatatcgaagaggggtggtctgcgaaggatttg
gacagacctcagatgcagagactgcttaaggatataaaaaaaggtaacatcgacattgtcctcgtatacaggttggata

ggctaactaggtccgttctggatctttatctgctcctgcaaacatttgagaagtacaacgtggcttttcgtagtgctacggagg
tatacgatacgtcaactgcaatgggccgtttattcattaccttagtggccgctctggcacaatgggagcgtgaaaacctggc
agagagggtaaaatttggaattgaacagatgattgacgagggcaagaagcccggaggtcacagtccctacggttataa
atttgataaggacttcaactgtactataatcgaggaggaagctgatgtcgtgcgtatgatttacaggatgtactgcgatggat
atggctaccgtagtatcgccgaccgacttaatgagttgatggtcaaacccaggatagccaaggaatggaatcataattcc
gttcgagatattcttacaaacgatatttatataggtacatacaggtggggtgataaagtagtccccaataatcatccgccaat
aatctccgaaactttgttcaaaaaagcacagaaggaaaaagaaaagcgtggcgttgaccgaaagcgagtcggaaag
tttctttttacaggactgttgcaatgcgggaattgcgggggacacaagatgcaagggcacttcgataagcgtgagcaaaa
gacttattaccgttgtactaaatgccaccgtatcacaaacgagaagaacatactagagccacttcttgacgagatacagtt
gttaattacgagtaaagagtactttatgagtaaatttagcgacagatacgatcaacaggaggttgtagacgtgagcgcgct
gactaaagaactggagaagataaagaggcagaaggagaagtggtacgatttgtatatggacgatcgaaatccgatcc
ccaaggaagagctatttgccaaaataaacgaattgaacaagaaagaagaggagatatatagtaagctaagtgaagtg
gaagaagataaagaaccggtagaagagaagtacaaccgtctctccaaaatgattgattttaaacaacagttcgaacaa
gcaaatgatttcacaaaaaaggaacttttgttctctatcttcgaaaagattgtaatctatagagagaaagggaaattaaag
aagatcacactagattatactttgaagtaa

Integrase 9 *A. thaliana* codon optimized sequence
atgaaggtcgctatatacaccagagtaagcactctggagcaaaaggagaaaggtcattctatagaagaacaggagag
gaagctcagagcatactccgatataaacgattggaaaattcataaagtgtatacagacgccggctattccggggcgaag
aaagaccgaccagcactacaagagatgttgaacgaaatagataacttcgatttggtgctcgtttataagctcgaccgact
cactcgttctgtcaaagacctacttgaaattctcgagctatttgagaataaaaatgtactctttcgatctgcgaccgaagtcta
tgataccacatcagccatgggacgacttttcgtgacgttggtgggcgcgatggcagagtgggaaagaacgactattcaa
gaaaggaccgcaatgggaagacgtgccagtgccagaaagggtcttgcgaaaacggtacctcctttctattatgacagg
gtcaatgacaagttcgtgcctaatgagtacaagaaagtgttgcgtttcgccgtggaagaagcgaagaaagggactagc
cttagagaaataacaatcaaactgaataacagcaagtacaaggcgccactcggaaaaaattggcacaggtccgtaat
cggaaacgcactcacctcacctgttgctagaggccatctagtatttggggacattttcgtggagaacactcacgaggcgat
aatatcagaagaggagtacgaggaaattaagcttagaatctctgagaagacgaactcaacaatcgttaaacataatgc
gatcttcaggagtaaactactatgtccaaactgcaatcaaaagcttaccctgaataccgttaaacatacacccaagaata
aggaagtgtggtattcaaagctatatttctgcagtaactgtaagaatacgaaaaataagaatgcttgtaacattgacgaag
gtgaggttttaaagcaattctataattatctaaagcaatttgatctgacttcctataaaatcgagaaccagccaaaggaaat
cgaagatgtgggtatcgacattgagaagttgaggaaagagcgagcgagatgccagacgctcttcattgaaggtatgatg
gacaaagatgaggcttttcctattatatcacgaatcgacaaggagatacacgagtatgagaaaaggaaagataacgac
aaagggaaaacattcaattatgaaaaaatcaagaactttaagtacagtcttctcaatgggtgggaactcatggaggatga
acttaagaccgagttcataaagatggcaatcaaaaatatacattttgaatatgtgaaagggataaaggggaagaggca
aaattctttgaagatcacgggcatagaattttattaa

Integrase 13 *A. thaliana* codon optimized sequence
atggctgtcggaatttacatacgagtgagtactcaggagcaggccagtgaggggcattccatagaaagccaaaagaa
aaaattggcgtcttactgcgagattcaggggtgggatgactataggttttatattgaggagggaatatccgggaagaatac
aaatcgtccaaagcttaaactccttatggagcatattgaaaagggtaaaattaatatactactcgtgtaccgattagaccgt
ctaacgaggtcagtaatcgatttgcacaagttattgaattttctgcaggaacacggttgcgccttcaagtccgcaactgaaa
cttacgacacaaccaccgcaaacggaaggatgagtatggggattgtatcactccttgcccagtgggagacggaaaata
tgagcgaacgtataaaactgaatctcgaacacaaagtactagtagagggggaaagggttggggctatcccctacggat
ttgatctaagtgatgacgaaaagctagtgaaaaatgaaaaaagtgcaattctgctggatatggtggagcgagtagaaaa
tggatggtccgtcaatcgaatcgtgaattacctgaatcttacgaacaatgaccgaaattggagtccgaatggcgtcctgcg
tctacttcgaaacccggcgctctacggggcgacccgttggaacgataagatagctgagaacacacacgaaggaattat
cagtaaagagaggttcaatagattgcagcaaatcctagcagatcgttctattcatcacaggcgtgatgttaagggtaccta
catttttcagggcgtattaagatgtccggtttgtgatcagacattgagcgttaacagattcatcaagaagcgaaaggatggc
acggagtactgtggcgttttgtatagatgccagccctgtattaaacaaaataaatacaatcttgcaattggcgaggcacgat
ttctgaaggctctcaacgagtatatgtccacggtggaatttcaaactgttgaggacgaggtcatccctaagaagagtgagc
gagagatgctggaaagtcaacttcaacagatagctagaaagagagaaaagtatcaaaaagcgtgggcttctgatttgat
gtctgacgacgagtttgaaaagcttatggttgagacccgagagacttacgatgaatgcaagcagaaactcgagtcttgcg
aggaccctatcaaaatcgacgaaacttacttaaaggagatagtttatatgttccatcaaaccttcaacgacctggagtccg
agaaacagaaggagttcatctcaaagtttattcgtacaatacgttacaccgtgaaggaacagcaaccaataaggccag
acaagagcaagactgggaaaggcaagcagaaagtcataattactgaggtagaattctatcaataa

phiC31 *A. thaliana* codon optimized sequence
atggacacatacgcgggtgcctatgacaggcagtcccgtgaacgtgaaaatagtagtgcggcttcccccgccacgcag
cgttccgccaatgaggacaaagcagcagacctccaacgagaggttgagcgagatgggggcaggttcaggttcgtagg
ccacttctctgaagcgcccggcacttccgcgttcggcaccgcggagagacccgagttcgagcgtatactaaatgagtgc
cgagcgggtcgactcaacatgataattgtatatgacgttagtaggttctccagactgaaagtaatggatgcaataccaata
gtatcagagcttctagccttgggcgttacgatagtgagcacccaggagggcgtattccgacaagggaacgttatggatctt
atacatctaattatgcgacttgatgccagtcacaaggaatcctccctcaagagcgccaagattcttgatactaagaaccta
cagagggagctcgggggttacgtcgggggaaaagccccgtacggttttgaacttgtcagtgaaaccaaggagattacg
aggaacgggaggatggtcaatgttgtcataaacaagttggcgcactcaactactccattgactggtccgttcgagtttgag
ccagacgtaattcgatggtggtggcgtgagatcaaaactcataagcatttaccttttaaaccgggcagccaggcggcaat
acaccccggaagcatcacaggcctgtgcaaacgtatggacgccgacgctgtgccgaccagaggcgagactattggg
aaaaagactgcctcctcagcatgggatcccgctacagttatgagaattttacgtgaccccagaatcgctggcttcgcagc
ggaagtgatttataaaaagaagccagatggcacacccacaacaaaaattgaggggtacaggatccagagagatcct
atcactttaaggccagttgagttggattgtggaccaataattgaacccgccgagtggtatgagcttcaggcctggttggatg
gaagaggtcgtggaaaaggcctcagccgtggacaggccatcctatctgctatggacaaattgtattgcgaatgcggcgc
ggtgatgacaagcaagcgaggcgaggaatctatcaaggacagctacaggtgtcgtagacgtaaagttgttgacccttca
gcacctggccagcatgagggtacgtgcaatgtctccatggcggcgttagacaagttcgtggctgaacgtatatttaacaa
aattcgacatgcagagggcgacgaggagactctagcgttactatgggaagccgcgcgtcgatttgggaagttaacgga
ggcgcctgaaaaaagcggagagagagctaatctcgtagccgagcgtgctgatgcactgaatgcgctagaggagctgt
atgaggaccgtgccgccggggcatacgatgggccggttggaaggaagcatttccgtaaacagcaagcggctctgaca
ctgcgtcagcaaggagccgaagagcgattagcggagctcgaagccgccgaggcgcctaaacttccactcgatcagtg
gttccctgaagacgcggacgccgatccgactgggcccaaatcttggtggggtcgagcatccgttgatgacaagcgtgtat
ttgtgggtctatttgtcgacaagatcgtagtgactaaatcaactacaggccgtggccagggcacgccaatcgagaaacgt
gcgagcataacatgggctaaaccacctacggacgacgatgaagacgatgcacaagatggaactgaggacgttgcag
catag

Bxb1 *A. thaliana* codon optimized sequence
atgagggccttagtcgtgatccgactatccagggtcactgatgccaccacatcaccagaaagacaactagagtcatgcc
agcaattgtgcgcacaaagaggatgggatgttgtaggcgttgcggaggacctagatgtatcaggtgccgtggaccccttt
gatagaaaacgaagaccaaacctagcgcgttggctcgcttttgaagagcaaccgtttgatgtgatcgtggcatacagggt
ggatagactaactaggagtatacgacacctacagcagttagtacattgggctgaggatcataagaagctagtggtctctg
caactgaggcccatttcgacacgaccaccccttttgccgcggttgtcattgcgttaatgggcaccgtagcacaaatggaac
tcgaggcaatcaaggaaagaaaccgaagcgcagcacactttaacattcgagccgggaagtaccgaggctctctgccc
ccttggggctatctcccgacgagggtggatggtgaatggagattagtaccagaccccgtccagagggaaaggattctag
aagtgtaccaccgagtggtggataatcacgaaccgctccacctcgtggcgcacgacttaaaccgtcgtggggtgctctct
ccaaaggactattttgcgcagcttcagggtcgtgaacctcaggggcgagagtggtccgctactgctttgaaacgtagtatg
attagcgaggcaatgttaggatatgctacgctaaacggcaaaaccgtccgagatgatgacggcgcgccgctagtacga
gctgaacctatactgacaagagaacagcttgaggctttacgtgcggagttagttaagactagcagggcaaaaccagca
gttagtacgccttcactgttactcagggttctgttttgcgcagtctgtggtgagcccgcgtacaaattcgctggtgggggcgt
aagcacccgcgttaccgttgtcgtagtatgggcttcccaaagcattgcgggaatggaaccgtggcaatggcggagtggg
acgctttctgcgaggagcaagtattggatctcctcggggatgctgaacgactggagaaagtgtgggtagctgggtctgatt
ctgctgtcgagctagcggaagtaaatgctgaactagtggacttaacgtccctcattgggtctccggcatatagagctggca
gccctcaaagggaagcccttgatgcgcgtatcgccgccctcgcagcgagacaggaagaattggagggctggaggct
aggccatcaggctgggagtggcgagaaacggggcagcgatttggtgattggtggagagagcaggatacggccgcta
aaaatacgtggcttaggagtatgaacgtgcgtctgacgtttgatgttcgtggtggtctcacacgaactattgactttggtgact
tgcaagaatacgagcagcacttacgattgggaagtgttgttgaacgtctacatacgggcatgagttag

References

1. Yang, L. *et al.* Permanent genetic memory with >1-byte capacity. *Nat. Methods* **11**, 1261–1266 (2014).
