# Supplementary material for: Genetic switches designed for eukaryotic cells and controlled by serine integrases
Source: Commun Biol. 2020 May 22;3:255. doi: 10.1038/s42003-020-0971-8 (PMC7244727; doi:10.1038/s42003-020-0971-8)
Supplement: Supplementary file 7 — Description of Additional Supplementary Files [file 42003_2020_971_MOESM7_ESM.pdf]

Description of Additional Supplementary Files

File Name: **Supplementary Data 1**
Description: HEK 293T cells aligned sequences. DNA cloned sequence reads we obtained after *egfp* inversion by Int activity in pSG plasmids in HEK 293T cells aligned with the expected flipped sequence.

File Name: **Supplementary Data 2**
Description: Bovine fibroblasts aligned sequences. DNA cloned sequence reads we obtained after *egfp* inversion by Int activity in pSG plasmids in fibroblasts aligned with the expected flipped sequence.

File Name: **Supplementary Data 3**
Description: *A. thaliana* protoplasts aligned sequences. DNA cloned sequence reads we obtained after *egfp* inversion by Int activity in pSG plasmids in protoplasts aligned with the expected flipped sequence.

File Name: **Supplementary Data 4**
Description: *A. thaliana* protoplasts aligned sequences. DNA cloned sequence reads obtained after CaMV 35S promoter inversion by Int activity in pSP plasmids in protoplasts aligned with the expected flipped sequence.

File Name: **Supplementary Data 5**
Description: PBMCs aligned sequences. DNA cloned sequence reads we obtained after *egfp* inversion by Int activity in pSG plasmids in PBMCs from three donors aligned with the expected flipped sequence.
